# Unveiling metabolo-genomic insights of potent antitumoral and antibiotic activity in *Streptomyces* sp. VB1 from Valparaíso Bay

**DOI:** 10.3389/fmicb.2024.1463911

**Published:** 2024-10-02

**Authors:** Néstor Serna-Cardona, Leonardo Zamora-Leiva, Eduardo Sánchez-Carvajal, Fernanda P. Claverías, Andrés Cumsille, Karla Alexa Pentón, Beatriz Vivanco, Alesia Tietze, Catherine Tessini, Beatriz Cámara

**Affiliations:** ^1^Laboratorio de Microbiología Molecular y Biotecnología Ambiental, Centro de Biotecnología DAL, Universidad Técnica Federico Santa María, Valparaíso, Chile; ^2^Millennium Nucleus Bioproducts, Genomics and Environmental Microbiology (BioGEM) Avenida España, Valparaíso, Chile; ^3^Laboratorio de Electroquímica y Química Analítica, Departamento de Química, Universidad Técnica Federico Santa María, Valparaíso, Chile; ^4^Centre for Antibiotic Resistance Research, University of Gothenburg, Gothenburg, Sweden; ^5^The Wallenberg Centre for Molecular and Translational Medicine, University of Gothenburg, Gothenburg, Sweden; ^6^Centre for Antibiotic Resistance research, University of Gothenburg, Gothenburg, Sweden

**Keywords:** marine *Streptomyces*, supercluster, BGCs, secondary metabolites, antimicrobial compounds, antiproliferative compound, molecular networking

## Abstract

*Streptomyces* sp. VB1, an actinomycete isolated from marine sediments in Valparaíso Bay, Chile, synthesizes antimicrobial and antiproliferative compounds. This study presents comprehensive metabolomics and comparative genomics analyses of strain VB1. LC-HRMS dereplication and Molecular Networking analysis of crude extracts identified antibiotics such as globomycin and daunorubicin, along with known and potentially novel members of the arylomycin family. These compounds exhibit activity against a range of clinically relevant bacterial and cancer cell lines. Phylogenomic analysis underscores the uniqueness of strain VB1, suggesting it represents a novel taxon. Such uniqueness is further supported by its Biosynthetic Novelty Index (BiNI) and BiG-SCAPE analysis of Gene Cluster Families (GCFs). Notably, two Biosynthetic Gene Clusters (BGCs) were found to be unique to VB1 compared to closely related strains: BGC #15, which encodes potentially novel anthracycline compounds with cancer cell growth inhibition properties, and BGC #28, which features a non-canonical configuration combining arylomycin, globomycin, and siamycin BGCs. This supercluster, the first described to consist of more than two adjacent and functional BGCs, co-produces at least three antimicrobial compounds from different antibiotic families. These findings highlight *Streptomyces* sp. VB1’s potential for discovering new bioactive molecules, positioning it as a promising candidate for further research.

## 1 Introduction

The bacterial genus *Streptomyces*, belonging to the phylum actinomycetota (Oren and Garrity, 2021), is renowned for its prolific production of bioactive compounds (Belknap et al., 2020). Bioprospecting efforts have highlighted strains isolated from unique and underexplored habitats, such as oceans (Sharma et al., 2019), as particularly divergent, with potential for novel natural product biosynthesis (Davies-Bolorunduro et al., 2021). Consequently, marine-derived actinomycetes have been extensively studied for their production of diverse natural products with various bioactivities (Sharma et al., 2019; Jagannathan et al., 2021).

The bioprospecting campaigns conducted by our research group led to the isolation of *Streptomyces* sp. VB1 from Valparaíso Bay, Chile, a port city characterized by high anthropogenic activity. Recent studies in this area have revealed the presence of new bacterial taxa at both the species and genus levels (Claverías et al., 2019, 2022). Previous investigations have highlighted the potential of *Streptomyces* sp. VB1 in producing bioactive compounds, including potentially novel anthracycline derivatives. Notably, crude extracts containing these compounds exhibited significant growth inhibition against clinically relevant model strains such as *Staphylococcus aureus* and *Listeria monocytogenes* (Cumsille et al., 2017).

It is well known that the synthesis of these bacterial natural products is coded in biosynthetic gene clusters (BGCs), which are groups of genes, physically clustered, that encode their biosynthetic pathway (Medema et al., 2015). These exhibit noteworthy diversity in their structural organization (Belknap et al., 2020), and consequently can produce a high variety of compounds.

Several bioinformatics tools have been developed to predict the presence of these BGCs in a genome and study them (Lee et al., 2020; Kenshole et al., 2021), generally based on the rules that govern the function of only a few of them. Recent studies have challenged the widely accepted canonical behavior of BGCs, revealing that in certain instances, the actual interactions within bacterial cells differ significantly from the established norms. Complex and dynamically regulated clusters, once deemed “rare,” are now recognized as more prevalent than previously believed (Ninomiya et al., 2016; Meyer and Nodwell, 2021). Given the potential niche for discovering new microorganisms in Valparaiso Bay, the exploration of *Streptomyces* sp. VB1, an isolated strain from that region, becomes particularly appealing. This strain has shown promise by demonstrating the production of potentially novel metabolites, making it a compelling subject for further investigation at the metabolomic and genomic levels, to elucidate its molecular traits, phylogeny, and biosynthetic capabilities potentially responsible for the forementioned bioactivities.

## 2 Results

### 2.1 Morphological and physiological properties of *Streptomyces* sp. VB1

**Figure 1:**
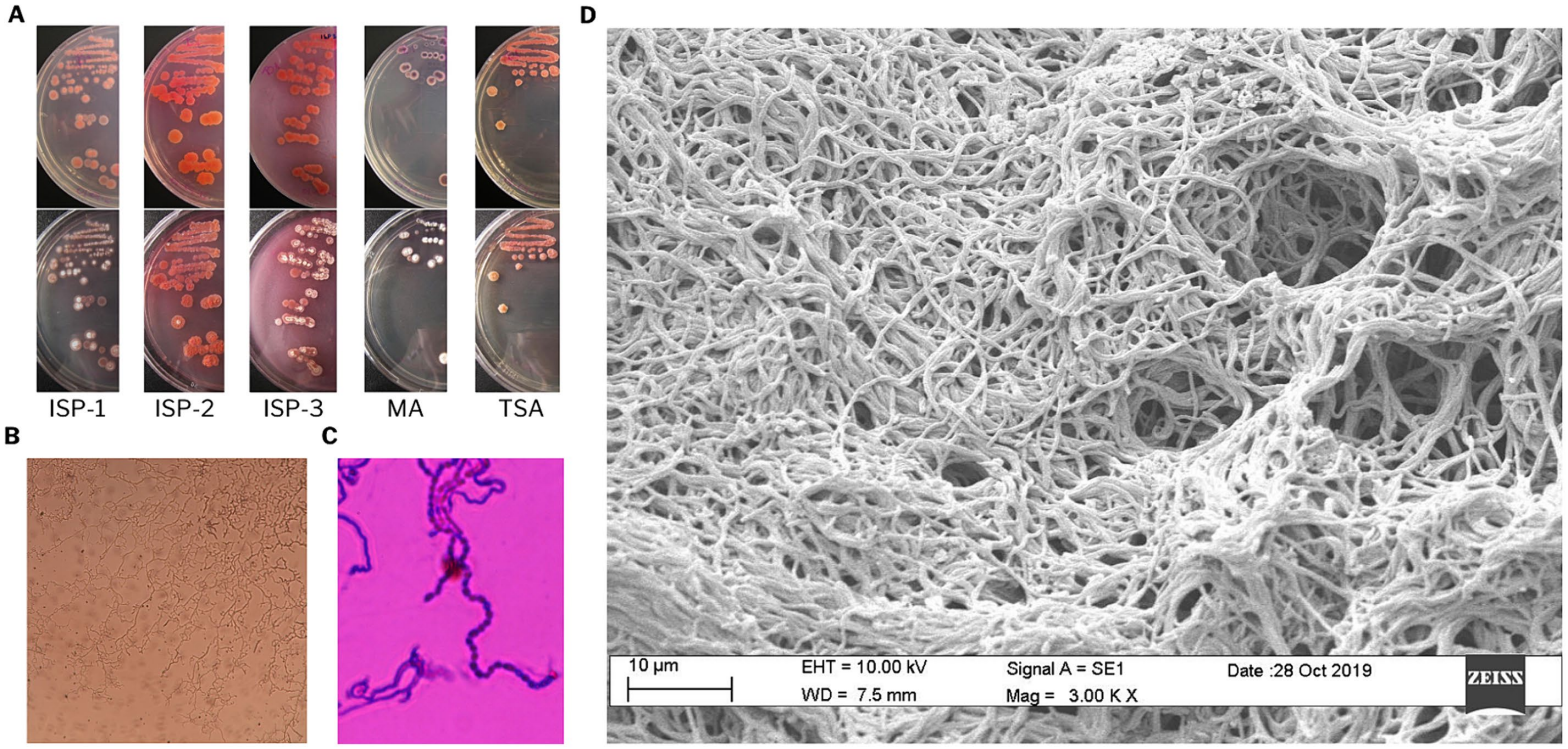
Morphological characterization of *Streptomyces* sp. VB1. **(A)** Cultural characteristics on ISP1, ISP2, ISP3, MA, and TSA media. **(B)** Mycelial characteristics, and **(C)** spore chain stained with crystal violet, observed under light microscopy at 100× magnification. **(D)** Scanning electron micrograph showing substrate mycelium.

To evaluate the potential of *Streptomyces* sp. VB1 to produce bioactive secondary metabolites, a comprehensive analysis encompassing morphological, physiological, genomic, and metabolomic characterization was assessed. Pink colonies were consistently observed in all media, with the most pronounced pigment formation and diffusion seen in ISP-2 and ISP-3 media (Figure 1A). Morphological observations of the *Streptomyces* sp. VB1 revealed the presence of extensively branched substrate mycelium and the formation of aerial mycelium, which differentiated into spore chains (Figures 1B–D). All tested sugars were utilized as the sole carbon source by *Streptomyces* sp. VB1; however, its characteristic reddish phenotype was observed in all plates except in media supplemented with mannitol, inositol, and xylose. Growth was observed within the temperature range of 15 to 35°C, with optimal growth occurring at 30°C, but not at 4 or 40°C. Additionally, *Streptomyces* sp. VB1 exhibited growth across a range of NaCl concentrations from 0 to 7% (w/v).

### 2.2 Biological and chemical analysis of *Streptomyces* sp. VB1 extracts

To explore VB1 strain potential for producing bioactive metabolites, fermentations were conducted using five distinct culture media, crude extracts were then evaluated for antimicrobial efficacy against five Gram-positive and Gram-negative model strains. LC-HRMS techniques and databases (such as PubChem, COCONUT, and Dictionary of Marine Natural Products) were utilized for compound identification through chemical dereplication.

The findings confirm the previously observed efficacy of VB1 extracts against *Staphylococcus aureus* (STAU) and *Listeria monocytogenes* (LIMO) model strains (Cumsille et al., 2017). Additionally, this effectiveness was evident across four extracts obtained from ISP1-ASW, ISP2-ASW, R5A, and SG media. Furthermore, a fractionation procedure was conducted on the extract produced in ISP1-ASW, resulting in six crude fractions. Among these fractions, F4 and F5 demonstrated selective inhibition against STAU and LIMO, respectively (Supplementary Table S1).

**Figure 2:**
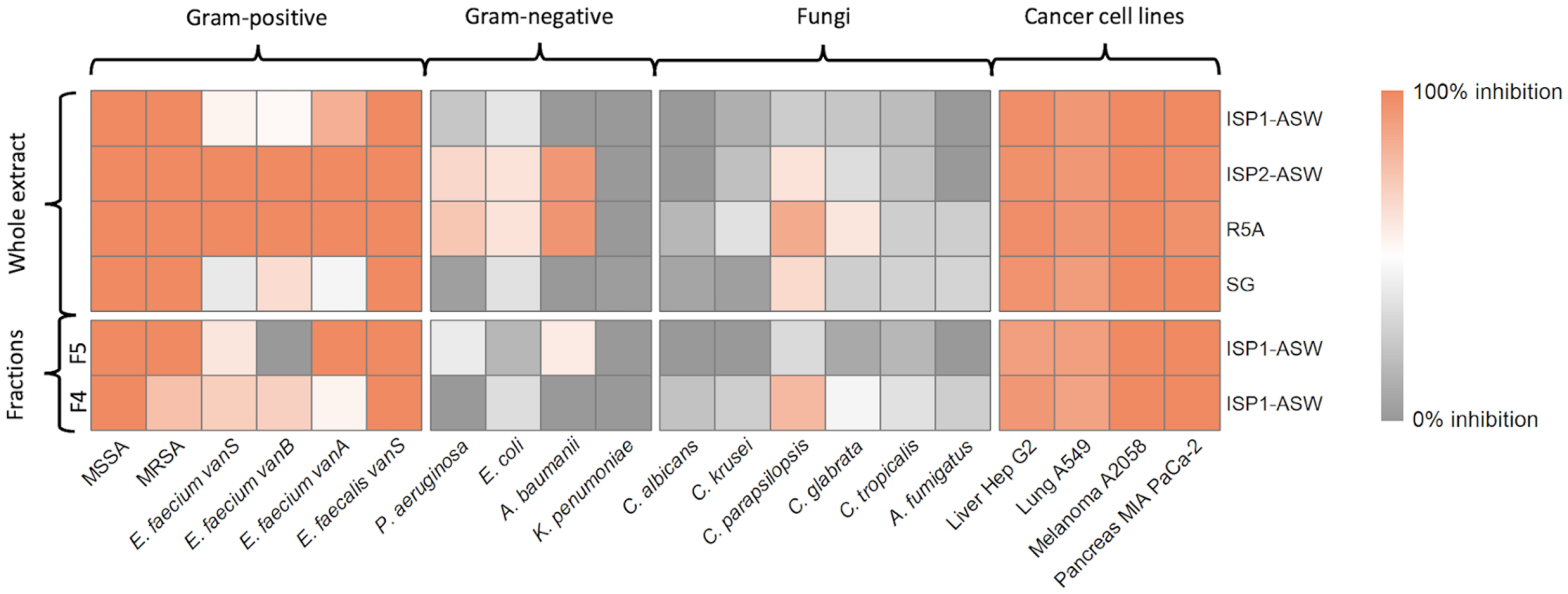
Bioactivity assays. Heatmap illustrates the percentage of growth inhibition obtained for each treatment. The culture media from which the extracts were derived are indicated on the right, and distinctions between whole extracts and their fractions, to the left. The bottom section indicates the microbial strains and cell lines used for testing, while the top section specifies their respective groups.

The bioactive crude extracts and fractions were subsequently assessed against a panel of pathogenic agents implicated in clinically relevant diseases, encompassing 10 bacterial, six fungi, and four cancer cell lines (Figure 2). The results for these extracts showed significant activity against at least 50% of the tested panel, demonstrating efficacy against all Gram-positive bacteria and cancer cell lines. Significantly, there was activity against *Acinetobacter baumanii*, a Gram-negative clinical pathogen, in extracts obtained from ISP2-ASW and R5A media.

The chemical dereplication (LC-HRMS) of crude extracts and fractions unveiled the presence of known bioactive compounds, including globomycin, arylomycin A4, and A5, effective against both Gram-positive and Gram-negative bacteria (Kiho et al., 2004; Roberts et al., 2011). Additionally, MKN 003B, known for its promising antifouling activity, was identified (Wang et al., 2014). The metabolic profiles of these extracts also revealed another group of compounds lacking matches within the explored databases, suggesting that VB1 may be synthesizing novel microbial metabolites.

### 2.3 Molecular networking analysis identifies potential new members of the arylomcyin and daunorubicin families

**Figure 3:**
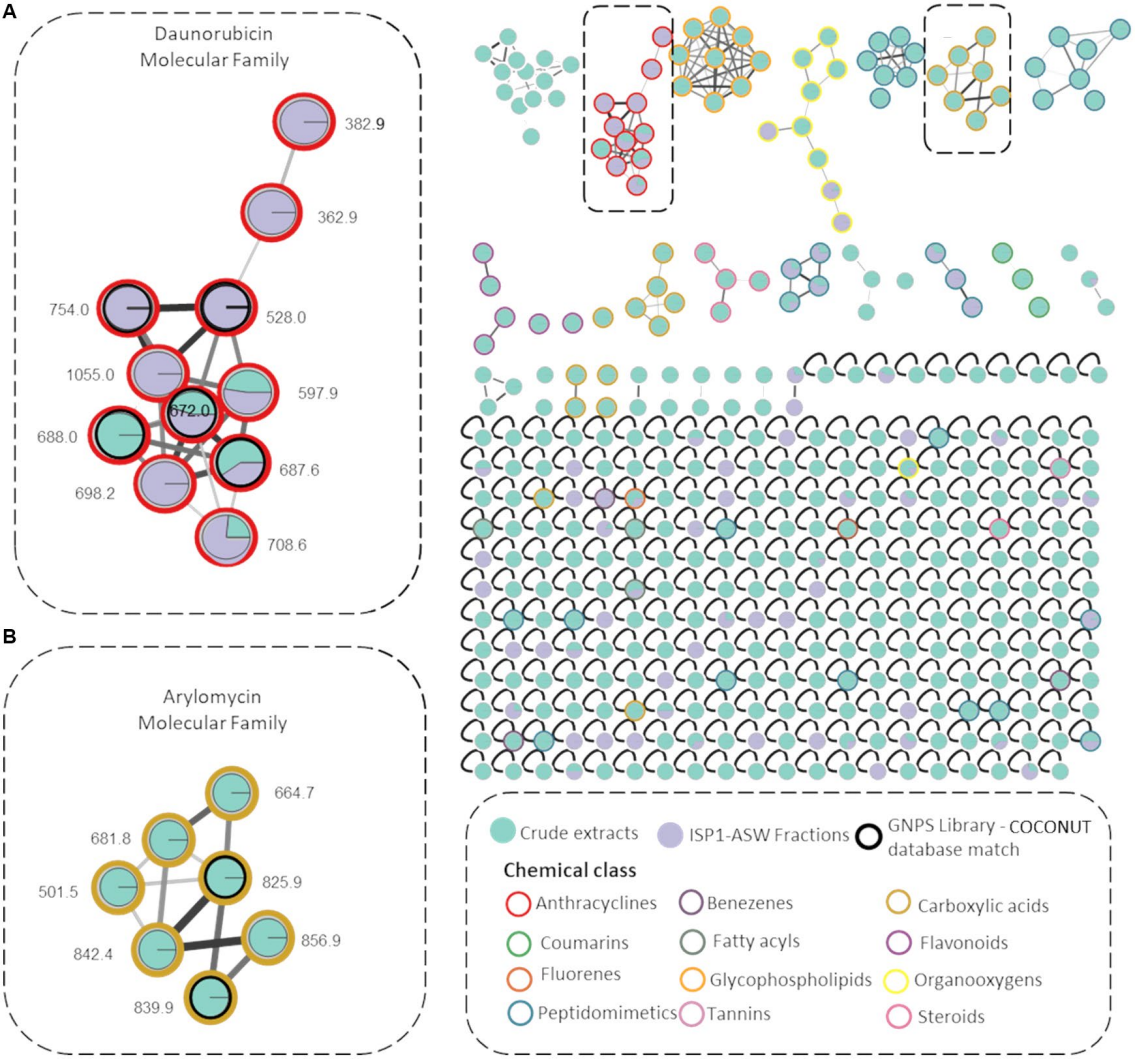
Molecular Networks of *Streptomyces* sp. VB1 extracts. Network illustrating 338 parent ions generated by *Streptomyces* sp. VB1 across four media (green nodes) and fractions F4 and F5 (purple nodes). Compounds identified by the GNPS library are enclosed in black outlined circles. Node border colors represent 12 annotated chemical class terms, assigned using the MolNetEnhancer workflow. Gray border nodes indicate parent ions that did not match any chemical class. The two highlighted molecular networks correspond to **(A)** Daunorubicin molecular family **(B)** Arylomycin molecular family.

To enhance the annotation of known molecules, a rapid dereplication process was achieved by creating a molecular network using the MS/MS data of all six microbial extracts and identifying the chemical class of the resulting molecular families (MFs). Utilizing the MolNetEnhancer workflow, 12 potential chemical classes were annotated within the molecular network (Figure 3). These are distinguished by colored borders within the nodes, with notable representations including anthracyclines (highlighted in red for the daunorubicin molecular family) and carboxylic acids and derivatives (highlighted in green for the arylomycin molecular family). Additionally, diverse MFs such as fatty acids, steroids, and glycophospholipids were observed. These components play pivotal roles not only in bacteria but also in a wide array of organisms, significantly contributing to development, survival, adaptation, and pathogenicity in various biological contexts (Manteca and Yagüe, 2018).

A molecular network consisting of 338 parent ions (nodes absent in media controls) was created, where 80% of the ions are only detected within the microbial crude extracts and 10% of ions were observed exclusively in F4 y F5 fractions. Also, 117 of these nodes were grouped within 25 MFs and 221 were singletons indicating that their fragmentation pattern did not correlate with any other parent ion of these. Also, 18 MFs comprise metabolite ions found exclusively within the crude extracts.

A molecular family encompassing seven distinct nodes including metabolites identified as arylomycins was observed (Figure 3), validated with the dereplicated ions *m/z* 825.0316 (arylomycin A2) and *m/z* 839.0421 (arylomycin A4) obtained from the chemical dereplication (Supplementary Figures S1A,B). The remaining five nodes potentially represent undiscovered arylomycins or their precursors. The analysis also revealed the presence of a daunorubicin molecular family, comprising 11 nodes primarily found within fractionated extracts. Within this network, five nodes were annotated using both the GNPS library and the COCONUT database (Sorokina et al., 2021), representing known anthracycline members of the daunorubicin family (Supplementary Figure S1C). Additionally, six nodes were not annotated as any known daunorubicin, thereby suggesting their classification as potential novel daunorubicin constituents. All dereplicated nodes are listed in Supplementary Table S2.

### 2.4 Genome sequencing and mining of biosynthetic gene clusters

To inspect its biosynthetic potential and gain better insights into its evolutionary relationships, the genome of *Streptomyces* sp. VB1 was sequenced. The final assembly showed a completeness of 99.2% and contamination of 1.19% according to CheckM (Parks et al., 2015) results. The complete genome of *Streptomyces* sp. VB1 contains a chromosome of 8,684,380 bp and a plasmid of 120,502 bp (Supplementary Figure S2A), and its general characteristics according to the NCBI Prokaryotic Genome Annotation Pipeline (PGAP) v6.3 (Tatusova et al., 2016), can be found in Supplementary Table S3. DeepNOG (Feldbauer et al., 2021) annotation performed through GenoVi (Cumsille et al., 2023) identified a total of 8,711 genes, grouped into 24 categories according to the COGs classification, indicating that 2,351 of them are involved in cellular processes and signaling, 1,715 in information storage and processing and 3,345 in metabolism. The remaining 1,300 of the predicted CDSs are poorly characterized, giving further evidence of the strain’s novelty potential (Supplementary Figure S2B).

Genome mining results of strain VB1 revealed a total of 34 putative secondary metabolite BGCs, according to antiSMASH results (Supplementary Table S4). Some of these BGCs have a 100% similarity with BGCs coding for the synthesis of SGR polycyclic tetramate macrolactam, AmfS, melanin, ectoine, coelibactin and a carotenoid. Furthermore, the complete BGCs responsible for the synthesis of desferrioxamine B, keywimysin, elaiophylin and coelichelin were identified with high similarity (75–99%) with known gene clusters, suggesting that *Streptomyces* sp. VB1 can produce these known compounds or novel analogs. Also, eight BGCs displayed moderate similarity (30–70%), while 12 displayed low similarity (1–20%) to known gene clusters, indicating a potential for the synthesis of novel analogs. The remaining four putative BGCs are cryptic and unknown, suggesting these might synthesize entirely novel molecules (Supplementary Table S4).

### 2.5 Phylogenomics-based comparative analysis

The fact that closely related strains can share genomic characteristics, and that many of these traits can be acquired through horizontal gene transfer (HGT), is widely acknowledged (Ochman et al., 2000). To determine the novelty of the biosynthetic capabilities observed both at the genomic and metabolomic levels in strain VB1, a comparison with other *Streptomyces* was performed, following a two-step phylogenomic tree-based comparative analysis. The first phylogenomic tree was generated through Orthofinder v2.3.11 using the curated genomes of each type strain within the *Streptomyces* genus as delineated in the actinobacterial tree, based on the previously constructed by Nouioui in the year 2018 (Nouioui et al., 2018).

#### 2.5.1 Phylogenomic insights suggest that *Streptomyces* sp. VB1 is potentially a new species

Our phylogenomic analysis (Supplementary Figure S3), revealed that *Streptomyces* sp. VB1 clustered along with *S. anulatus* JCM 4721^T^, *S. griseus* DSM 40236^T^*, S. durocortorensis* RHZ10^T^, *S. rubiginosohelvolus* JCM 4415^T^ and *S. puniceus* NRRL B-2895^T^. Notably, *S. anulatus* JCM 4721^T^ emerges as the closest taxonomic neighbor to *Streptomyces* sp. VB1. However, when considering the 95–96% ANI species delineation threshold (Richter and Rosselló-Móra, 2009), it is possible to observe that *Streptomyces* sp. VB1 does not exhibit an ANIb value exceeding 95% when compared to any other validly described *Streptomyces* with available genomes (Supplementary Figure S4).

**Figure 4:**
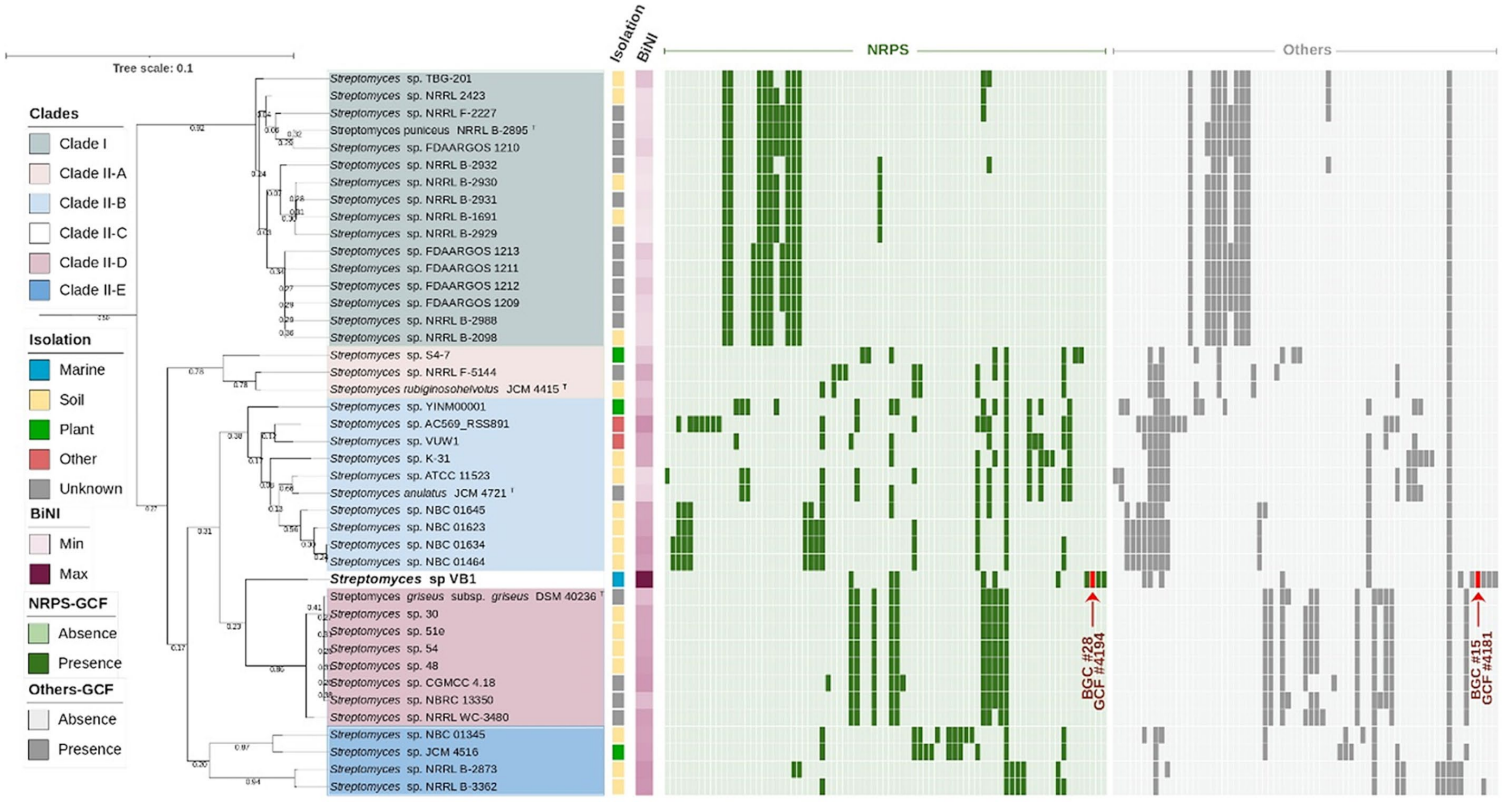
VB1’s closest-neighbors tree. Phylogeny of *Streptomyces* strains with ANIb >90% against *Streptomyces* sp. VB1 filtered dataset of *n* = 41 strains of the species *S. griseus*, *S. anulatus*, *S. rubiginosohelvolus*, *S. durocortorensis* and *S. puniceus* from the RefSeq database (Supplementary Table S6). The evolutionary distance is depicted as a bar, considering 0.01 substitutions per amino acid position. The tree’s clades, isolation source and BiNI values are color-coded as indicated in the figure legend. GCFs originated using BiG-SCAPE v1.1.5, are shown in a presence/absence matrix, where NRPSs GCFs are depicted in green and Other GCFs in gray.

To extend the understanding of strain VB1’s biosynthetic novelty, a second phylogenomic tree with the closest neighbors was constructed, and later complemented by a comparative analysis of BGCs. The *Streptomyces* type strains with higher ANIb values (88–95%, Supplementary Figure S4) were selected for this analysis, and all high-quality genomes of strains documented under the *S. griseus*, *S. anulatus, S. durocortorensis, S. rubiginosohelvolus,* and *S. puniceus* species deposited within RefSeq were used (Figure 4). The architecture of the phylogenomic tree is configured by two large clades, clade I where the type strain of the *S. puniceus* species is located; and clade II where the VB1 strain is located. Additionally, clade II is divided into 5 subclades: clade IIA constituted by strains that group with *S. rubiginosohelvolus* JCM 4415^T^, clade IIB conformed by strains related to *S. anulatus* JCM 4721^T^, clade IIC where only strain VB1 is located, clade IID with strains related to *S. griseus* subsp. *griseus* DSM 40236^T^, and finally, clade IIE with some strains deposited in RefSeq under *S. griseus* and *S. anulatus* species. However, due to the distance with respective type strains, it is suggested that they may not belong to those species. Both phylogenomic trees (Supplementary Figure S3; Figure 4), in accordance with the ANIb results (Supplementary Figure S4), suggest that strain VB1 may constitute a new species, or alternatively, a known species whose genome remains non sequenced or unavailable in RefSeq within the *Streptomyces* genus.

#### 2.5.2 Comparative genomics reveals outstanding biosynthetic capabilities in *Streptomyces* sp. VB1

To assess the biosynthetic distinctiveness of *Streptomyces* sp. VB1, we computed its Biosynthetic Novelty Index (BiNI) (González-Salazar et al., 2023). Utilizing BiNI scores, we systematically ranked all closely related *Streptomyces* strains (Figure 4), revealing that strains from clade II exhibit higher BiNI values compared to those in clade I. Specifically, strains *S. griseus* DSM 40236^T^ (BiNI = 667.1), *S. rubiginosohelvolus* JCM 4415^T^ (BiNI = 658), *S. anulatus* JCM 4721^T^ (BiNI = 595.9), and *S. puniceus* NRRL B-2895^T^ (BiNI = 567.5) all demonstrated significantly lower potential for novelty, when compared to strain VB1. Notably, strain VB1 displayed the highest BiNI value (BiNI = 984.4), indicating the presence of the most distinctive BGCs with novel biosynthetic potential compared to other strains.

To enhance the scrutiny of BGC diversity, we constructed a BGC network employing BiG-SCAPE, depicted as a presence/absence matrix alongside the second phylogenomic tree (Figure 4). Remarkably, among the BGCs identified, a singular BGC (GCF 1568) is ubiquitously present across all strains. This BGC has been delineated as a siderophore, as per the antiSMASH classification with approximately 3% of its genes exhibiting homology to ficellomycin (Supplementary Table S4). Furthermore, this BGC does not exhibit any discernible resemblance to any previously characterized BGC within the MiBIG database (Manteca and Yagüe, 2018).

Distribution patterns of “NRPS” and “Others” GCFs, depicted in green and gray, respectively, (Figure 4), reveal that certain clades, notably clade I (16 strains) and clade IID (8 strains), exhibit a more uniform distribution of GCFs. This suggests a significant role of vertical transfer mechanisms (inheritance) in the BGC acquisition process within these strains (Ziemert et al., 2014). In contrast, clade IIB, comprising 10 strains, displays irregular patterns in the distribution of both NRPS and other GCFs, with certain GCFs observed in only a limited number of strains. Such distribution patterns suggest that these BGCs may have been acquired through HGT, a mechanism involved in genomic diversification (Seipke, 2015).

Among the examined strains, besides *Streptomyces* sp. VB1, another 11 exhibited the presence of at least one unique BGC not assigned to any NRPS or Others GCF, all of which belong to clade II (Supplementary Table S12). Though only 239 of the total 1705 BGCs (~14%) that were analyzed in this study are in contig edges and possibly fragmented, it is the case for 12 out of the 40 BGCs (30%) deemed as unique, which can affect the GCFs distribution, and not necessarily reflect a real uniqueness (Supplementary Table S12). In that sense, strain *Streptomyces* sp. AC569_RSS891, grouping in clade IIB, stood out with the highest number of unique BGCs, boasting 5 out of its 17 ‘NRPSs’ GCFs (~29%), and 3 out of its 13 ‘Other’ GCFs (~23%). However, both values are potentially overestimated due to BGC fragmentation, as 5 out of these 8 BGCs (62.5%) are located in contig edges (Supplementary Table S12). In contrast, no overestimation due to BGCs fragmentation was observed for the next two strains demonstrating a significant presence of unique BGCs. *Streptomyces* sp. S4-7, with 4 out of its 8 ‘NRPS’ GCFs (50%) and 1 out of its 9 ‘Others’ GCFs (~11%), and *Streptomyces* sp. VB1, featuring 4 out of its 10 ‘NRPS’ GCFs (40%) and 3 out of its 12 ‘Others’ GCFs (~33.3%) as unique.

Throughout this investigation, we have placed particular emphasis on two distinct GCFs, 4,181 and 4,194, exclusive to the VB1 strain in contrast with the rest of the strains in this study. The former corresponds to GCF classified as ‘Other’ and labeled as BGC #15 (Supplementary Table S8), potentially producing new antitumor compounds from the anthracycline family (Supplementary Figure S5). The latter, BGC #28 (Supplementary Table S4) in the antiSMASH prediction, a ‘supercluster’ comprised of three different antibiotic-producing BGCs (arylomycin, globomycin, and siamycin I, as illustrated in Supplementary Figure S6B) not previously described, as later verified by a remote cblaster advanced search. Notably, this supercluster may possess co-regulatory mechanisms governing its production. The activities of these three antibiotics are potentially synergistic, as they collectively impede cell wall formation in both Gram-positive and Gram-negative bacteria (Roberts et al., 2007; Vogeley et al., 2016; Tan et al., 2019).

The antiSMASH analysis indicates that 33% of the BGCs identified in the VB1 strain exhibit a low degree of similarity with those deposited in the MIBiG database. Many of these BGCs are unique to the VB1 strain compared to closely related taxa. Additionally, the VB1 strain displays a notably higher BiNI, highlighting its distinctive biosynthetic capabilities not only within its broader phylogenomic context but also in comparison to the nearest documented strains. These findings underscore the importance of further investigating these specific BGCs present in the genome of the VB1 strain.

### 2.6 Biosynthetic potential to produce novel antimicrobial and antitumor analogs

**Figure 5:**
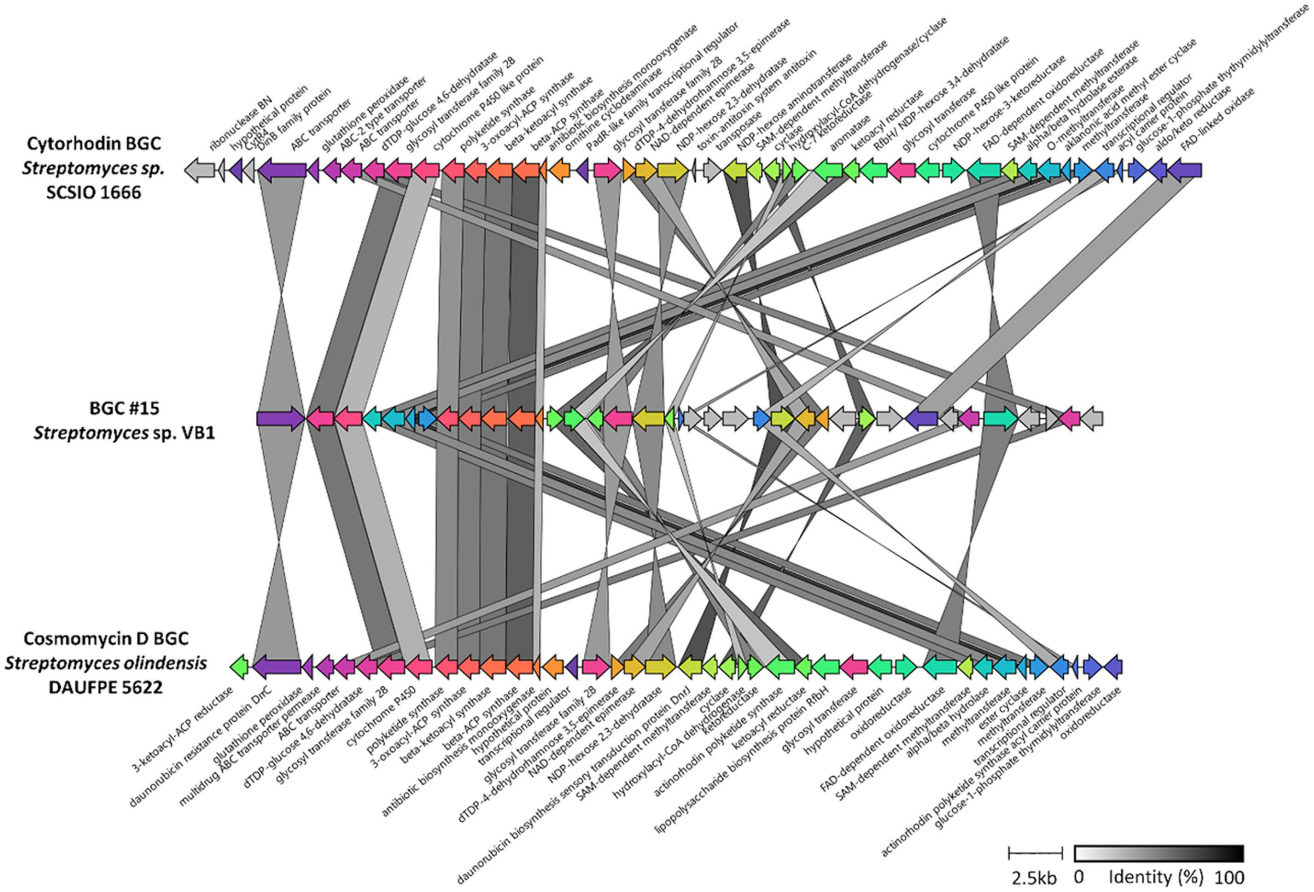
Structure of BGC #15 versus other anthracyclines BGCs. Comparison of the 80,592–58,217 section of BGC #15 against the two most similar BGCs, cythorhodin (BGC0001568) and cosmomycin D (BGC0001074). Genes are colored according to the presence of potential homologs in the other clusters, while for those in gray, no similar genes were found. Predicted products for each gene are shown for cythorhodin and cosmomycin D BGCs. Identity percentage between matching genes is shown in greyscale-colored links. BGC #15 and that of cosmomycin D are reversed for better visualization.

**Figure 6:**
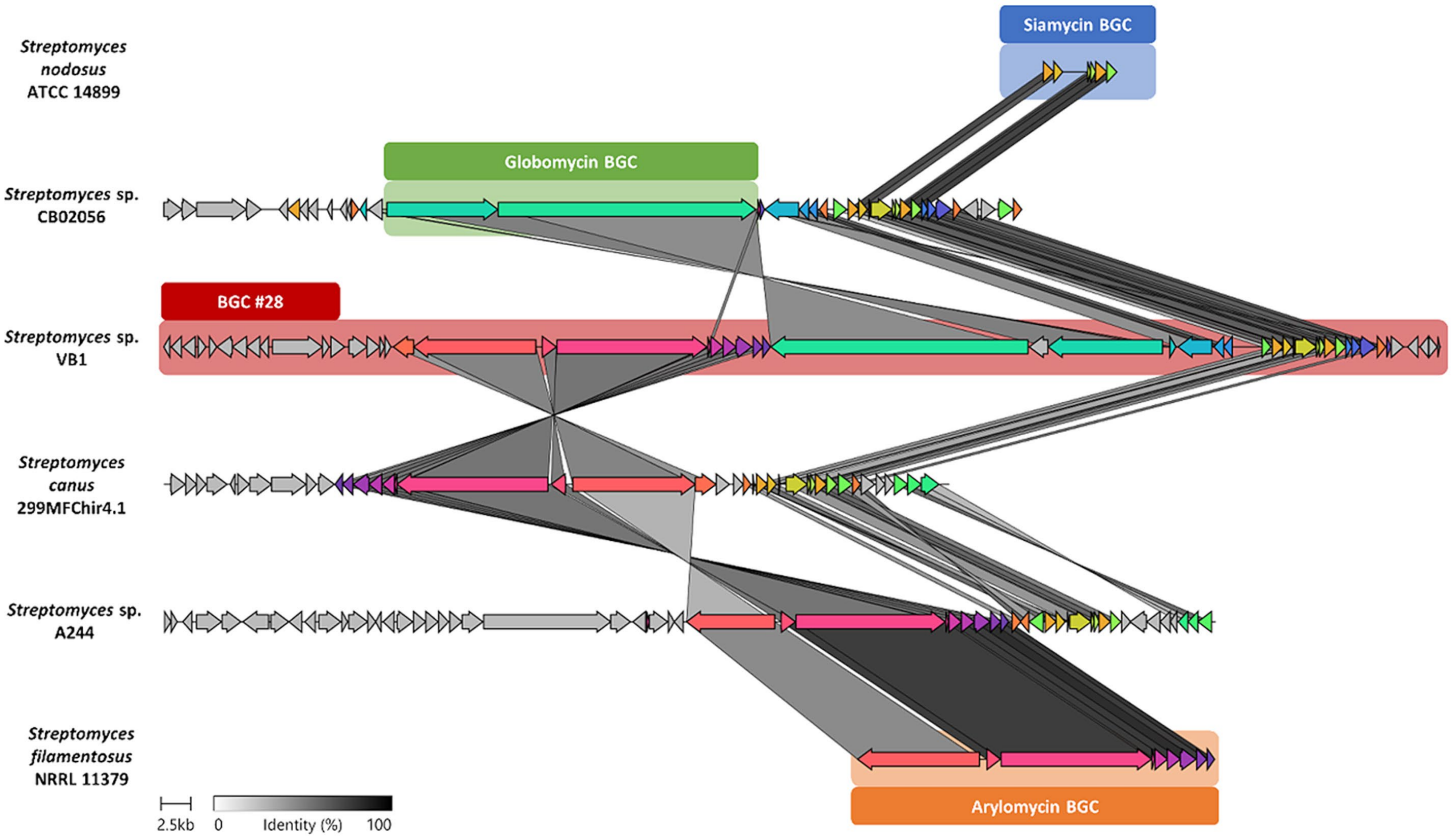
Structure of BGC #28 versus other similar BGCs. Comparison of the 21,425–104,757 sections of BGC #28 against BGCs, siamycin (BGC0001920) and arylomycin (BGC0000306), as well as that of globomycin, and those found by cblaster in other *Streptomyces* strains. Genes are colored according to the presence of potential homologs in the other cluster, while for those genes in gray, no similar genes were found. Identity percentage between matching genes is shown in greyscale-colored links.

To conduct a thorough analysis of the unique genetic components that give rise to the exclusive BGCs of the VB1 strain, a comparative gene-to-gene study was undertaken. Results from the analysis of BGCs #15 (Figure 5) and #28 (Figure 6) are presented in detail.

#### 2.6.1 Insights into BGC #15

Regarding BGC #15, the analysis revealed the presence of genes with a high similarity percentage with BGCs responsible for synthesizing compounds within the anthracycline class (Supplementary Figure S5A). This includes a complete similarity with doxorubicin BGC (BGC0000218) and similarities with known anthracycline BGCs documented in the MIBiG repository (Kautsar et al., 2020). These include a 50% match with genes from cytorhodin (BGC0001568), a 62% match with genes from cosmomycin D (BGC0001074), and a 60% match with genes from cinerubin B (BGC0000212) (Supplementary Figure S5B). Previous chemical dereplication results with this strain suggested that the antimicrobial activity in its crude extracts could be partially attributed to the presence of anthraquinones (Cumsille et al., 2017), and molecular networking also indicated the existence of molecules from this family in the bioactive extracts (Figure 3). Consequently, a detailed comparison was conducted between BGC #15 and two significant KnownClusterBlast matches (Figure 5), demonstrating that despite sharing a similar structure and a considerable number of seemingly homologous genes, the level of identity between these genes is only moderate (indicated by greyscale).

#### 2.6.2 Insights into the supercluster BGC #28

KnownClusterBlast results for BGC #28 (Supplementary Figure S6B) uncovered the presence of all genes coding for the synthesis of both siamycin (BGC0001779) and arylomycin (BGC0000306), well known bioactive molecules (Roberts et al., 2007; Tan et al., 2019). Furthermore, a detailed exploration of the remaining genes, located between those coding for siamycin I and arylomycin, shows some similarity to known NRPS-like BGCs reported in the MIBiG repository. This BGC contains two NRPS genes, which would synthesize a cyclohexa depsipeptide; OH133_33795 gene with four adenylation domains (PKS-NMe-Leu, Ile, Ser, Gly), and OH133_33815 gene with just one adenylation domain (Thr) as shown in the “module view” generated by the antiSMASH analysis (Supplementary Figure S7).

Findings from the chemical dereplication of organic extracts derived from *Streptomyces* sp. VB1 (Supplementary Figure S9), demonstrated its capacity to produce the globomycin antibiotic. Considering the similarity of the structure of globomycin, and the predicted molecule putatively synthesized by the NRPS modules in this specific BGC, it was hypothesized that BGC #28 encompasses a supercluster, that is, two or more BGCs that are often adjacent to each other (Meyer and Nodwell, 2021). To evaluate the distribution of this BGC across the *Streptomyces* genus, a comprehensive cblaster search against all the *Streptomyces* data in the RefSeq protein database was conducted. These results revealed that only one *Streptomyces* strain, CA-278952, contained a highly similar cluster in its genome (Supplementary Figure S8), for which the production of globomycin was recently reported (Oves-Costales et al., 2023). The CA-278952 strain was isolated from a soil sample at the Dílar riverbank in Granada, Spain (Oves-Costales et al., 2023), and was later revealed in this study to be closely related to the VB1 strain, with an ANIb 99.95%. Additionally, three other strains were found to contain at least two of the three sub-clusters comprising the proposed supercluster. These strains, *Streptomyces* sp. A24, *Streptomyce*s sp. CB02056, and *Streptomyces canus* 299MFChir4, were phylogenomically more distant when compared to the VB1 strain, exhibiting ANIb values of 81.07, 79.13, and 80.92%, respectively. For these results, a detailed comparison between these BGCs and those of reference (MIBiG) was constructed using clinker (Figure 6), revealing a moderate level of sequence conservation and slight variations in architecture.

Four regulators were found within the genes of BGC #28 according to the antiSMASH predictions, two displaying significant similarity to *mslG* (histidine kinase sensor) and mslR2 (LuxR family DNA-binding response regulator) present in the siamycin BGC. The remaining two belong to the sigma-70 family RNA polymerase sigma factor and the MarR family transcriptional regulator, intriguingly showing no similarity to the transcriptional regulators of globomycin and/or arylomycin. The abundance of regulators within this supercluster is noteworthy; however, this observation may be attributed to intricate cross-regulation mechanisms, a phenomenon previously documented in other BGCs (Romero-Rodríguez et al., 2015; McLean et al., 2019), especially because the MarR family is known for the regulation of antibiotic synthesis, such as avermectin (Guo et al., 2018).

### 2.7 Detection of globomycin, arylomycin and siamycin

The co-production of the compounds–globomycin, arylomycin, and siamycin–in crude extracts from R5 medium was evaluated using mass spectrometry. For identifying these antibiotics, we referenced the molecular ions obtained through chemical dereplication provided by the Medina Foundation: globomycin (*m/z* 656,7,), arylomycin A4 (*m/z* 839,7), and arylomycin A5 (*m/z* 858,7). Additionally, siamycin (*m/z* 2.164,2), identified through genomic comparative analysis, was referenced to the molecular mass of siamycin I (2163.5 g/mol) from the PubChem database. The simultaneous detection of the three antibiotic families constituting the supercluster was confirmed by observing the molecular ion of each antibiotic in the metabolic profile, as depicted in Supplementary Figure S9, affirming their presence in the R5 extract.

## 3 Discussion

Marine Actinomycetota represent a prolific reservoir of bioactive compounds, with most of these originating from the singular genus *Streptomyces*, drawing significant attention from the pharmaceutic market due to their remarkable capacity for generating unusual chemical structures. In this study, a novel Actinomycetota strain is described, *Streptomyces* sp. VB1. This strain, producer of three co-expressed antibiotics (globomycin, arylomycin, siamycin) and novel anthracycline compounds, was isolated from a marine sediment sample collected from Valparaíso Bay, Chile (Cumsille et al., 2017). According to the phylogenomic analysis in our study, strain VB1 is highly likely to be a novel taxon within the *Streptomyces* genus (Supplementary Figures S3, S4; Figure 4).

The analysis of secondary metabolite biosynthetic novelty revealed that the VB1 strain exhibited the highest novelty index among closely related strains (Figure 4). This observation may be attributed to the influence of oligotrophic ecosystems, such as marine environments, which promote the emergence of novel chemical compounds (Cumsille et al., 2017). Notably, research by González-Salazar et al. introduced the BiNI metric and highlighted that Actinomycetota, such as *Lentzea* sp. CC55 (BiNI = 1,008) and *Actinokineospora* sp. PR83 (BiNI = 1,049), isolated from the Cuatro Ciénagas Wetland in the Coahuila Desert, Mexico–a unique ecosystem characterized by low phosphorus availability and high heavy metal concentrations–exhibited higher BiNI values compared to *Lentzea* (24 genomes, average BiNI = 439.2) and *Actinokinespora* (17 genomes, average BiNI = 451.6) strains from more nutrient-rich environments. Additionally, wetland *Streptomyces* strains showed significantly higher novelty compared to reference *Streptomyces* genomes, with BiNI values up to three times greater (González-Salazar et al., 2023).

The biosynthetic uniqueness of strain VB1 is demonstrated in our study by the presence of two distinct BGCs, which are exclusive to VB1 and absent in closely related strains. A comprehensive analysis of the VB1 genome revealed a supercluster, BGC#28, which includes genes required for the biosynthesis of globomycin, arylomycin, and siamycin. The concept of biosynthetic superclusters–groups of BGCs within the same genomic region that allow for coordinated regulation and co-production of metabolites with potential synergistic action—is increasingly intriguing yet relatively underexplored (Meyer and Nodwell, 2021). Archetypal examples include the β-lactam supercluster in *Streptomyces*, which contains BGCs for cephamycin C and clavulanic acid. This antibiotic pair exhibits synergistic activity by combining the β-lactam antibiotic cephamycin with the β-lactamase inhibitor clavulanic acid (Ward and Hodgson, 1993). More recently, a supercluster of rapamycin and actinoplanic acid has also been discovered in *S. rapamycinicus* (Mrak et al., 2018). The synergy of these antibiotics arises from targeting two proteins crucial for the cell cycle, growth, and synthesis of essential cellular components in fungi. Rapamycin inhibits TOR complex 1 (TORC1) signaling, while actinoplanic acid is a potent and selective competitive inhibitor of Ras farnesyl-protein transferase, which is involved in TORC1 activation (Mrak et al., 2018). Additionally, other examples of synergistic antibiotics produced by superclusters have been identified in more distantly related bacterial *phyla* (Horsman et al., 2017; Arp et al., 2018). There are also superclusters with atypical architectures where genes coding for two chemically distinct compounds are scattered across the biosynthetic region. Examples include the pristinamycin (Mast et al., 2011) and anthrabenzoxocinone (Morshed et al., 2021) superclusters, which exhibit complementary antibiotic activity. Pristinamycin, a type of streptogramin, is biosynthesised by a mosaic-type BGC and inhibits cell growth by targeting the 50S ribosomal subunit. This results in bactericidal potency 10 to 100 times greater than the sum of their individual effects (Reissier and Cattoir, 2021). Recent hypotheses suggest that exploring these synergistically co-produced antibiotics represents a promising approach to combat antimicrobial resistance, enhancing the effectiveness of treatments by amplifying inhibition of bacterial growth and reducing the likelihood of resistance development (Meyer and Nodwell, 2021).

According to literature, the three supercluster-associated compounds described in this work (Figure 6), target the same cellular component, the bacterial cell wall, of both Gram-positive and Gram-negative bacteria. Arylomycins are a class of natural-product antibiotics known for inhibiting bacterial type I signal peptidase (*SPase*), a Ser-Lys dyad protease responsible for removing N-terminal signal sequences from preproteins following their translocation across the cytoplasmic membrane (Smith and Romesberg, 2012), whereas globomycin competitively inhibits the lipoprotein signal peptidase II (LspA), which is absent in eukaryotes and is considered an attractive target for new antibiotics development (Vogeley et al., 2016). Additionally, siamycin-I inhibits cell wall biosynthesis by binding to lipid II, an essential precursor for peptidoglycan biosynthesis (Tan et al., 2019). This fact, along with the unusual genomic arrangement of the BGC#28, strongly suggests that these compounds may exhibit a synergistic antimicrobial mechanism, possibly reflecting the evolutionary preservation of their genomic linkage within the ‘supercluster’.

This strategy, so conserved across the domains of life, considers that BGCs could evolve through the successive combination of smaller sub-clusters (Medema et al., 2014), so it is not rare to think that complete BGCs with specific functions can evolve to be together in a ‘supercluster’. The presence of the arylomycin, siamycin I and globomycin BGCs has been reported across the *Streptomyces* genus, either with the presence of only one BGC (*S. nodosus* ATCC 14899, siamycin; *S. filamentosus* NRRL 11379, arylomycin), or with two of the three BGCs (*Streptomyces* sp. A244, arylomycin and siamycin; *Streptomyces* sp. CB02056, globomycin and siamycin; *Strepomyces canus* 299MFchir4.1, arylomycin and siamycin) or all three BGCs clustered (*Streptomyces* sp. VB1 and *Streptomyces* sp. CA-278952) (Figure 6). This co-occurrence of BGCs that are members of the proposed ‘supercluster’ in phylogenetically distant strains suggests that each of these has been specifically maintained and optimized by selective pressure (Supplementary Figure S3). This affirmation is in concordance with recent experimental analysis of the inhibitory activity between arylomycin G0775 (synthetic derivative) and globomycin, which confirmed that mutant strains of *Staphylococcus aureus* resistant to arylomycin G0775 were inhibited by the joint action of these antibiotics, demonstrating that inhibitors targeting nonessential enzymes can be used to overcome resistance mechanisms, which could be an evolutionary reason to place two (or more) BGCs together (Stone et al., 2023).

To our knowledge, BGC#28 of *Streptomyces* sp. VB1 is the first described supercluster consisting of more than two adjacent and functional BGCs, each contributing to the co-production of at least three antimicrobial compounds from different antibiotic families, potentially with synergistic activity.

Additionally, VB1 extracts demonstrated significant inhibition of four cancer cell lines: Hep G2 liver, A549 lung, A2058 melanoma, and MIA PaCa-2 pancreas (Figure 2). Previous dereplication analysis showed the presence of baumacyin A1, A2, and benastatin A and B, recognized cytotoxic compounds of the anthracycline family (Cumsille et al., 2017). Anthracyclines are structurally characterized by an anthraquinone-based fused tetracyclic ring system, typically linked to an amino-sugar (Malik and Müller, 2016). Besides usually exhibiting antibiotic activity against Gram-positive bacteria (Hulst et al., 2022) and even potentiating the activity of some other antibiotics against Gram-negative bacteria (Cox et al., 2014), anthracyclines are more commonly studied for being among the most effective and widely commercialized treatments to fight many types of cancer, including leukemia, lymphoma, breast, stomach, uterine, ovarian, bladder, and lung cancers (Martins-Teixeira and Carvalho, 2020). These compounds can be glycosylated with a variety of carbohydrates, and to date, at least 5 different types of glycosides have been described for such glycosylations, which usually occur at the C-7 of the first non-aromatic ring, but in some cases, also at C-10. Biological activity of anthracyclines depends essentially on their glycosylation patterns (Fujiwara et al., 1985; Szafraniec et al., 2016). However, commercial anthracyclines have a main side effect, cardiotoxicity, which considerably limits their use (McGowan et al., 2017). Therefore, hundreds of analogs of natural and synthetic origin have been evaluated as potential chemotherapeutics to find compounds capable of replacing these drugs (Martins-Teixeira and Carvalho, 2020). Molecular network analysis revealed a network related to the daunorubicin molecular family. Notably, four of the 11 nodes in the network (*m/z* 597.9, *m/z* 698.2, *m/z* 708.6, and *m/z* 1,055.0) did not correspond with any recorded anthracycline in natural product databases, suggesting their potential as new anthracyclines.

A comparative genomic analysis of the closest anti-tumor members of the daunorubicin family–cytorhodin, cosmomycin D, and cinerubin B–revealed a common characteristic: these BGCs encode enzymes that are likely responsible for adding two trisaccharide chains, presumably linked to the C-7 and C-10 positions, with the primary distinction being the type of carbohydrate attached. Furthermore, a detailed comparison between BGC #15 of *Streptomyces* sp. VB1 and the cytorhodin and cosmomycin D BGCs (Figure 5), which included manual curation of each gene via BLAST, indicates a structural similarity with the cytorhodin biosynthesis pathway. However, the gene identity levels are only moderate (as depicted in greyscale), suggesting potential variation in the molecules synthesized by BGC #15. Additionally, two genes identified in the cosmomycin D BGC, *cosG* and *cosK*, have homologous counterparts in the cytorhodin BGC, encoding glycosyltransferases responsible for the addition of trisaccharides to the molecule’s rings (Garrido et al., 2006). Both genes also have homologs in BGC #15, suggesting that anthracyclines produced by this strain could also have two chains of trisaccharides attached to their structure. We also identified novel genes within BGC #15, highlighted in gray in Figure 5 and listed in Supplementary Table S13, which exhibit no homology to any genes from known anthracycline BGCs. Based on predictions from NCBI’s PGAP, these genes potentially encode a class I SAM-dependent methyltransferase (OH133_10805), an acyl-CoA dehydrogenase (OH133_10815), a cytochrome P450 (OH133_10840), glyoxylase CFP32 (OH133_10845), and an SDR family NAD(P)-dependent oxidoreductase (OH133_10850). The first two enzymes are involved in the synthesis of nitro-sugar D-kijanose of kijanimicin, one of the most highly functionalised sugars in nature (Al-Mestarihi et al., 2013). These genes show homology to *kijD1* (62% identity, 100% coverage) and *kijD3* (56% identity, 97% coverage) respectively.

The remaining three genes are implicated in the modification of the structural core of specialized metabolites. The OH133_10840 gene is homologous to the *makC2* gene, which hydroxylates the sesterterpenoid core during the biosynthesis of maklamicin (Daduang et al., 2015) (31% identity, 97% coverage). The OH133_10845 gene shows homology to the glyoxalase CFP32 gene (30% identity, 64% coverage), which plays a role in the methylglyoxal detoxification pathway, converting this highly toxic compound into lactic acid (He and Moran, 2011). Lastly, OH133_10850 shares similarity with the HitM1 gene, which is involved in the biosynthesis of hitachimycin (Kudo et al., 2015) (34% identity, 96% coverage); HitM1 is responsible for modifying the macrolactam skeleton of hitachimycin, specifically oxidizing the hydroxyl group at C-11. This set of genes may confer structural variations to the anthracyclines produced by *Streptomyces* sp. VB1, as depicted in the molecular networking analysis (Figure 3). Such variations could lead to the discovery of novel analogs, positioning this strain as a promising candidate for in-depth study in the search for potential chemotherapeutic drugs.

The comprehensive analysis of *Streptomyces* sp. VB1 has unveiled its distinctive genomic and metabolomic capabilities, particularly attributed to the unique features of BGCs #15 and #28. These findings suggest the potential for the development of structurally novel compounds with antimicrobial and chemotherapeutic properties. The unprecedented identification of a supercluster producing three antibiotic peptides with potential synergistic antimicrobial activity signifies a promising approach to combat antimicrobial resistance, potentially broadening the efficacy and spectrum of existing antibiotics. Moreover, the capability of *Streptomyces* sp. VB1 to co-produce three synergistically active antibiotics, points it as a candidate for further studies on synthetic biology and scale-up strategies for industrial production. Furthermore, the study of this marine-derived *Streptomyces* strain from Valparaíso Bay, Chile, not only advances our understanding of the biosynthetic mechanisms underlying secondary metabolite production but also contributes significantly to the broader landscape of comparative genomics and metabolomics, facilitating the discovery of novel bioactive compounds.

## 4 Materials and methods

### 4.1 Strain isolation, phenotypic and morphological characterization

*Streptomyces* sp. VB1 was isolated from a shallow marine sediment sample collected from the coast of Carvallo Beach (33°1′8.69′′S 71°38′32.38′′W) in Valparaíso, Chile, (Cumsille et al., 2017) and deposited in the CCUG Culture Collection (Culture Collection University of Gothenburg, Sweden) under the number CCUG 74964.

Morphologic analysis of strain VB1 was accomplished using the following media: yeast extract-malt extract agar (ISP2), oatmeal agar (ISP3) (Shirling and Gottlieb, 1966), Marine agar 2,216 (MA, Difco), and tryptic soy agar (TSA, Difco). Two microscopy techniques were used to evaluate cell morphology. First, light microscopy (Axio Lab.A1, Zeiss) was performed using the coverslip technique described by Williams and Cross (1971). Samples were obtained from a R5A medium culture incubated for 21 days at 30°C. And second, a scanning electron microscopy (model ABC; Zeiss), which was performed on cultures grown on ISP2 medium at 30°C for 3 days.

To assess carbon source utilization, the strain was cultured in modified ISP2 medium by replacing glucose with one of the following carbohydrates, obtaining a final concentration of 1% (w/v) of the tested compounds: cellulose, L-arabinose, D-fructose, D-glucose, I-inositol, D-mannitol, rhamnose, sucrose and D-xylose. To evaluate the optimal growth temperature, different cultures were tested at 4, 15, 20, 25, 30, 35, 37, 40, and 45°C on ISP2 agar medium for 10 days. NaCl tolerance was evaluated at various concentrations (1, 3.5, 5, 7, and 10% NaCl w/v) using Luria Bertani (LB) agar.

### 4.2 Fermentation, crude extracts preparation and fractionation

For the preparation of crude extracts, *Streptomyces* sp. VB1 was fermented during 7 days at 30°C, 200 rpm, in a 50 mL volume ISP1 (5 g/L tryptone, 3/L g yeast extract), ISP2 (10/L g malt extract, 4 g/L yeast extract, 4 g/L glucose), R5A (Fernández et al., 1998), SM19 (Malmierca et al., 2018) and a modified-SG medium, with soytone peptone –instead of soytone – and with no added glucose. ISP1 and ISP2 media were prepared with artificial sea water (ASW) (i.e., ISP1-ASW and ISP2-ASW). Afterwards, cells were separated from the supernatant by centrifugation at 5000 rpm for 10 min. Supernatants were extracted twice in a decantation funnel using ethylacetate (EtOAc) in a 1:1 (v/v). The process is repeated twice for each sample. The recovered organic phase is then almost completely evaporated with a rotary evaporator and the remaining extract dried on a vacuum concentrator. Dried extracts were dissolved in dimethyl sulphoxide (DMSO) (20% *v*/*v*) to the final concentration of 4 μg/mL, excepting R5A and SM19 media, where 1,8 and 3 μg/mL concentrations were used, respectively.

Scale-up fermentation and fractionation protocol is briefly described as follows: 5 mL of *Streptomyces* sp. VB1 pre-inoculum in TSB was transferred to 40 flasks of 250 mL containing 50 mL of ISP1-ASW medium for a total volume of 2 L of fermentation. The cultivation conditions included a temperature of 30°C, agitation at 200 rpm for 7 days. At the end of the fermentation, the culture was centrifuged, and the supernatant was extracted with EtOAc as described in the previous paragraph. A total of 200 mg of dry extract of strain VB1 in ISP1-ASW was fractionated using a semi-preparative HPLC system (Waters corporation) equipped with a Waters 1,525 binary HPLC pump and a DAD detector, using an ISERA ISAspher 300–10 C18 column (250 × 30 mm) with a flow rate of 10 mL/min. The mobile phase consisted of solvent A (ACN) and solvent B (water), both with 0.01% trifluoroacetic acid. The gradient composition started with a linear decrease of solvent A from 10 to 90%, and a linear increase of solvent B from 90 to 10%, over 100 min. These conditions were then maintained for 20 min with 95% solvent A and 5% solvent B, followed by a 20-min return to the initial conditions. A total of seven fractions were finally obtained. Dried fractions were dissolved in DMSO (20% *v*/*v*) to the final concentration of 4 μg/mL.

### 4.3 Antimicrobial activity

The antibacterial activity of crude extracts and fractions were produced and corroborated as previously was described (Cumsille et al., 2017) and results are shown in Supplementary Table S1.

The bioactive crude extracts and fractions were chosen for additional investigation regarding their potential as antibiotic and antiproliferative agents against clinically relevant pathogenic bacteria and fungus strains and cancer cell lines, all sourced from Fundación Medina.

Antibacterial assays were performed using the following bacterial strains: *Staphylococcus aureus* ATCC 29213 (MSSA), Methicillin resistant, *Staphylococcus aureus* MB5393 (MRSA), *Enterococcus faecalis* 14,492, *Enterococcus faecium vanS* 144,754, vancomycin resistant *Enterococcus faecium vanB* 176,308, vancomycin resistant *Enterococcus faecium vanA* 15,167, *Pseudomonas aeruginosa* MB5919, *Escherichia coli* ATCC 25992, *Acinetobacter baumanii* MB5973 and *Klebsiella pneumoniae* K6 (*K. quasipneumoniae* subsp. *similipneumoniae* ATCC 700603). Methods were applied as described by Audoin et al. (2013). Briefly: MRSA and *Enterococcus* strains were cultured on Brain heart infusion (BHI) medium, while MSSA, *K. pneumoniae*, *P. aeruginosa*, *E. coli* and *A. baumanii* strains were cultured on Mueller Hinton II (MHII) medium. All agar plates were incubated at 37°C for 24 h, and using a single colony, a liquid culture was inoculated and fermented overnight at 37°C and 150 rpm, except for *E. coli,* grown for 2 h. Liquid cultures were then adjusted to the following OD600: 0.310 for MRSA, 0.300 for *E. faecium vanA* 15,167, 0.160 for *E. faecium vanB* 176,308, 0.180 for *E. faecium vanS* 144,754, 0.111 for MSSA, 0.450 for *K. pneumoniae*, 0.350 for *P. aeruginosa*, 0.450 for *E. coli*, and 0.350 for *A. baumanii.* These were then diluted 1:100 until further use. As controls, antibiotic dose–response curves of the following compounds were used: vancomyc in (320 μg/mL) for MSSA; aztreonam (20 μg/mL) for *E. coli*, *P. aeruginosa*, and *A. baumanii*; and gentamycin (320 μg/mL) for *K. pneumonia*.

Moreover, antifungal activity was assessed against the following six fungal strains: *Candida albicans* ATCC 64124, *Candida krusei* ATCC 6258^T^, *Candida parapsilopsis* ATCC 22019^T^, *Candida glabrata* ATCC 2001^T^, *Candida tropicalis* ATCC 750^T^ and *Aspergillus fumigatus* ATCC 46645. *Candida* strains were grown on RPMI agar media at 37°C for 24–48 h, single colonies were resuspended on RPMI broth until reaching an OD660 of 0.25–0.28, and then diluted 1:10 until further use (Audoin et al., 2013). Assays against *A. fumigatus* was performed using resazurin and as previously described by Monteiro et al. (2012).

Moreover, 96-well plates were used for antimicrobial activity assays, where 90 μL of each strain were mixed with 5 μL of *Streptomyces* sp. VB1 strain crude extract resuspended on DMSO 20% w/v. Furthermore, 90 μL of each culture media mixed with 5 μL of DMSO 20% w/v were used as blanks. Likewise, 90 μL of each previously adjusted inoculum were mixed with 5 μL of DMSO 20% w/v for use as growth control. Previously mentioned antibiotics were employed as internal controls in antibacterial assays. A dose–response curve was constructed by combining 90 μL of each previously adjusted inoculum, with a four-point serial dilution of 10 μL of the respective antibiotic. Plates were incubated at 37°C for 24 h. A Tecan Ultraevolution spectrophotometer was used to measure OD 600 nm, at time zero (before incubation) and at 24 h. Growth inhibition was calculated using the following Equation 1 (Audoin et al., 2013):


(1) $$\begin{array}{l} \mathrm{Inhibition}\;\left(\%\right)\\ =\left[1-\frac{\left(T_{f\;\mathrm{Sample}}-T_{0\;\mathrm{Sample}}\right)-\left(T_{f\;\mathrm{blank}}-T_{0\;\mathrm{blank}}\right)}{\left(T_{f\;\mathrm{Growth}}-T_{0\;\mathrm{Growth}}\right)-\left(T_{f\;\mathrm{blank}}-T_{0\;\mathrm{blank}}\right)}\right]x100 \end{array}$$

### 4.4 Antiproliferative assays

The antiproliferative activity was evaluated using the MTT Tetrazolium reduction method (Mosmann, 1983), in four cancer cell lines: Hep G2 liver, A549 lung, A2058 melanoma, and MIA PaCa-2 pancreas. The cells were cultured in 96 well plates at a density of 1,000,000 cells per plate. The plates were incubated at 37°C, with 90% humidity and 5% CO_2_, for 24 h. Afterwards, the medium was changed, and the cells were diluted with media to a final DMSO concentration of 0.5%. The plates were then incubated for 72 h under the same conditions mentioned earlier. The first and last columns of the 96 well plates were reserved for controls and standards. The first column contained alternating positive and negative controls, with Methyl methanesulfonate (MMS) 8 mM as the positive control and DMSO 100%/20% as the negative control. The standard was established using a dose–response curve with doxorubicin at 5 mM, employing an 8-point serial dilution (1/3). After incubation, the plates were washed using a multidrop with 100 μL of PBS x1 per well. Subsequently, the wells were treated with an MTT solution. The MTT solution was prepared by diluting MTT in PBS x1 (5 mg/mL) and then in MEM culture medium without phenol red (0.5 mg/mL). The solution was added to the wells at a volume of 100 μL per well using a multidrop, and the plates were incubated for approximately 3 h at 37°C. The supernatant was then removed, and 100 μL of DMSO 100% was added to each well to dissolve the formazan precipitates. Finally, the absorbance levels were measured using the Wallac 1,420 VICTOR2TM at a wavelength of 570 nm, and the resulting data were analyzed using Genedata Screener® software, which provided the percentage of growth inhibition.

Growth inhibition percentages for both antibacterial and antiproliferative assays were used to create a heatmap for graphical representation using R v4.1.3 (R Core Team, 2023).

### 4.5 UPLC-MS/MS dereplication analysis

Chemical dereplication was performed on bioactive crude extracts (10 μg/mL), as well as on bioactive fractions (F4 and F5) (4 μg/mL) using liquid chromatography-high resolution mass spectrometry (LC-HRMS). Experiments were carried out using an HPLC 1200 Rapid Resolution (Agilent, Santa Clara, CA, United States) coupled to a MaXis HRMS (Bruker, Billerica, MA, USA). A Zorbax SB-C8 column (Agilent, Santa Clara, CA, USA) was used for separation (2.1 × 30 mm, 3.5 μm) with a constant flow rate of 0.3 mL/min. Mobile phase consisted of solvent A, MQ-H_2_O:ACN 90:10 and solvent B, MQ-H_2_O:ACN 10:90; both with 13 mM ammonium formate and 0.01% TFA. Gradient composition started with a linear decrease of solvent A from 90 to 0% and a linear increase of solvent B from 10 to 100% in 6 min. Afterwards, conditions were maintained for 2 min with 0% solvent A and 100% solvent B, followed by a recovery of initial conditions for an additional 2 min. HRMS was operated in positive mode with a spray voltage at 4 kV, 11 L/min at 200°C capillary temperature and 280 Kpa pressure at the nebulizer. Absorbance was measured at 210 nm. Data-dependent MS2 scans were obtained using collision-induced dissociation (CID) with an energy of 35 eV and activation time of 30,000 ms for the first, second, and third most intense peaks. Molecular formulae, UV spectra, accurate masses, and retention times (RT) were obtained for crude extract predominant components for their potential identification (De la Cruz et al., 2017). For the identification of known compounds, molecular formulae and accurate masses were obtained for the predominant components of the crude extract, and comparison of their retention times and masses were used as criteria to search for candidates in the Fundación MEDINA in-house database. Where no match was obtained, a complementary search in the Dictionary of Natural Products of Chapman and Hall was performed. Additionally, the monoisotopic masses, retention times and molecular formulae of metabolites predicted from VB1 genome and found in crude extracts of VB1 (i.e., Globomycin, arylomycin, siamycin, and anthracycline-like compounds) are listed in Supplementary Table S2.

### 4.6 Molecular networking

Mass spectra data was processed using MZmine v2.53 (Pluskal et al., 2010) freeware for peak detection, deconvolution, deisotoping, filtering, alignment, and gap filling. Parameters used involved an *m/z* tolerance of 0.015, a 1.00E3 intensity for peak detection, a minimum time span and RT tolerance of 0.1 min and a noise level set at 1.00E2 for mass detection.

A molecular network using the MS/MS data from crude extracts was created using the GNPS (Wang et al., 2016) online workflow.^1^ First, data was filtered by removing all fragment ions within +/− 17 Da of the *m/z* precursor and by choosing the top six fragment ions in the +/− 50 Da window throughout the spectrum. Precursor ion mass and MS/MS fragment ion tolerances were set to 2.0 Da and 0.5 Da, respectively. A network was then created in which nodes are connected if they have a cosine score above 0.7 and more than six matched peaks. Furthermore, connections between nodes are maintained only if the cosine score is within the first top 10 hits of the respective similar node. Finally, the maximum size of a molecular family (MF) was set to 100 nodes and the ones with a lower scoring edge below this threshold were removed. Spectra in the network were searched against GNPS’ spectral libraries, which were filtered as stated above for input data. A match between network and library spectra was considered when presenting a cosine score above 0.7 and at least six matched peaks. Cytoscape (Su et al., 2014) v3.6.1 was employed for molecular network visualization, where each node corresponds to a consensus spectrum and each edge represents a cosine similarity score between nodes.

To identify additional members of the Daunorubicin and Arylomycin families produced by the VB1 strain, we analyzed the molecular networks of these compounds from our Molecular Networking data. We extracted the ion masses of each node in the networks and compared them with entries in the COCONUT and PubChem databases. Matches between the masses indicated potential known members of these families. To enhance the chemical structural information in the molecular network, information from *in silico* structure annotations from GNPS Library Search was incorporated into the network using the GNPS MolNetEnhancer workflow^2^ on the GNPS website (Ernst et al., 2019). The annotations of the different chemical classes were made using the chemical ontology software ClassyFire (Djoumbou Feunang et al., 2016). The molecular networking analysis can be found here: https://gnps.ucsd.edu/ProteoSAFe/status.jsp?task=804381f36e904c53aa4eaf3412eb670e.

### 4.7 Genome sequencing, assembly, and annotation

The genomic DNA of *Streptomyces* sp. VB1 was extracted using a Wizard® Genomic DNA Purification Kit (Promega Corporation, Madison, WI, United States) according to the manufacturer’s instructions. The obtained genomic material was later tested for concentration and contamination using a NanoDrop Spectrophotometer (Thermo Fisher Scientific Inc.), for quantification using a Qubit Fluorometer (Thermo Fisher Scientific Inc.) and for integrity with an agarose gel electrophoresis, ultimately being stored at 4°C until further use. Sequencing was accomplished using the PacBio Single Molecule Real Time RS II platform (Macrogen Inc.) with one SMRT cell.

The resulting reads, as delivered by the service provider, yielded 881,619,739 bp distributed in 111,993 reads with an average length of 7,872 bp. The yielded reads were filtered using Filtlong v0.2.0 (Wick, 2017) (minimum length: 1,000 bp, minimum mean quality: 90%), and then used for a *de novo* assembly by Flye v2.4.1 (Kolmogorov et al., 2019) with default parameters. A preliminary assembly comprising 3 contigs, one of 8,656,701 bp and two small contigs of 120,502 bp and 27,579 bp, was obtained and later used for phylogenomic analysis. RFPlasmid v0.0.18 (van der Graaf-Van Bloois et al., 2021) was used to evaluate the possible plasmidic character of the 3 contigs in such assembly, identifying only one of them as plasmidic. The remaining two contigs, classified as non-plasmidic (8,656,701 bp and 27,579 bp), were further used as input for a reference-guided scaffolding to RagTag v2.1.0 (Alonge et al., 2021). The genome used as reference was that of the phylogenomically closest strain with highest assembly level available from RefSeq, *Streptomyces anulatus* YINM00001 (Liu et al., 2022).

The resulting final assembly, comprised by two contigs (linear chromosome and plasmid), was subjected to QUAST (Gurevich et al., 2013) and CheckM (Parks et al., 2015) for quality assessment, and annotation of gene functions was accomplished applying the NCBI Prokaryotic Genome Annotation Pipeline (PGAP) v6.3 (Tatusova et al., 2016). The complete genome sequence of *Streptomyces* sp. VB1 was deposited in GenBank under the accession numbers CP110539 and CP110540, for chromosome and plasmid, respectively. The general characteristics of the genome of *Streptomyces* sp. VB1 can be found in Supplementary Table S3. A circular genome representation was generated using GenoVi (Cumsille et al., 2023), which integrates DeepNOG (Feldbauer et al., 2021) to further annotate the corresponding Clusters of Orthologous Groups (COGs) categories for each gene (Supplementary Figure S2). Finally, the genome mining of secondary metabolite BGCs was accomplished using the antiSMASH v6.1.1 (Blin et al., 2021) webserver (Supplementary Table S4).

### 4.8 Phylogenomic analysis against *Streptomyces* type strains

For phylogenomic analysis, we selected 104 type strain genomes from the *Streptomyces* genus (Supplementary Table S5), initially based on the actinobacterial tree constructed by Nouioui in 2018 (Nouioui et al., 2018). Subsequently, we updated the dataset with the latest available genomes closely related to *Streptomyces* sp. VB1. *Kitasatospora xanthocidica* JCM 4862^T^ was used as an outgroup.

All genomes were downloaded from NCBI RefSeq FTP server (104 entries as of 5th July 2023). Selected *Streptomyces* genomes were annotated using Prokka v.1.14.5 (Seemann, 2014) to further construct a phylogenomic tree using Orthofinder v2.5.4 (Emms and Kelly, 2019). For tree construction, Diamond (Buchfink et al., 2021) aligner was employed for orthogroup retrieval, MAFFT (Katoh and Standley, 2013) for multiple sequence alignment, and FastTree (Price et al., 2010) for approximate maximum-likelihood (ML) tree inference. The final tree was modified using iTOL web version v6.81 (Letunic and Bork, 2021). To complement tree topology and for phylogeny inference, an additional ANIb analysis was performed using FastANI v0.2.12 (Jain et al., 2018).

### 4.9 Comparative genomics and biosynthetic analysis of *Streptomyces* strains

The biosynthetic capacity of the *Streptomyce*s sp. VB1 strain was assessed through comparative analysis with phylogenetically related strains. According to the phylogenomic tree construction, the closest related *Streptomyces* species were selected for further analysis. All strains deposited under *Streptomyces anulatus*, *Streptomyces griseus*, *Streptomyces rubiginosohelvolus, Streptomyces durocortensis* and *Streptomyces puniceus* species were downloaded from NCBI RefSeq database through the FTP server (with the only exception of *S. puniceus* strains, which are deposited under the *Streptomyces californicus* name) (Supplementary Table S6). All genomes were subjected to quality filters (Undabarrena et al., 2021) using the CheckM v1.0.18 (Parks et al., 2015) and Quast v5.0.2 (Gurevich et al., 2013)^.^ Genomes with less than 200 contigs (larger than 500 bp), completeness >95%, and contamination <2% were selected for further analysis. Strain downloads and quality analyses were performed with an automated pipeline written in Python, deposited at: https://github.com/LeonardoZamoraLeiva/network_workflow. Then, a second phylogenomic tree was constructed as described in the previous section. An additional ANIb analysis was performed using PyANI v0.2.12 (Pritchard et al., 2015). The resulting tree was visualized and annotated through the iTOL v6.8.1 web tool (Letunic and Bork, 2021).

For each of the genomes, annotations were used to predict BGCs using standalone antiSMASH v6.1.1 (Blin et al., 2021), which were then grouped into Gene Cluster Families (GCFs) using the BiG-SCAPE v1.1.5 software (Navarro-Muñoz et al., 2019). For network construction, a raw distance cutoff value of 0.3 was used. A Python script^3^ was used to transform the output into a presence/absence matrix of GCFs per strain, which was graphically represented as a heatmap using the iTOL 6.8.1 (Letunic and Bork, 2021) web tool.

Additionally, genomes were used for predicting their BGCs using the antiSMASH v7.0 (Blin et al., 2023) web tool as required for comparison with the BiG-FAM (Kautsar et al., 2021) database and calculation of their BiNI (González-Salazar et al., 2023), a recently proposed metric to rapidly rank strains according to the novelty of their BGCs in contrast with the diversity of GCFs found in the BiG-FAM (Kautsar et al., 2021) database. Though a more recent version of antiSMASH is used for this analysis, the number of BGC types that it can predict is less than 15% higher than the previous version (Blin et al., 2023), and only slight changes in BGC predictions are observed between results. Thus, overall biosynthetic novelty is not greatly affected and still serves the goal of the study.

Two BGCs from *Streptomyces*. sp. VB1 were selected for further analysis. For BGC #28, a remote cblaster (Gilchrist et al., 2021) homologous gene cluster search was performed against the Refseq protein database (Entrez term: Streptomyces [organism]). The most relevant results from such search were further subjected to a clinker (Gilchrist and Chooi, 2021) gene cluster comparison. As for the BGC #15, a direct clinker comparison with the two most similar gene clusters according to antiSMASH’s (Blin et al., 2023) KnownClusterBlast results was performed. All cblaster and clinker analyses were run through the CompArative GEne Cluster Analysis Toolbox (CAGECAT) web server (van den Belt et al., 2023).

### 4.10 Determination of co-production of globomycin, arylomycin and siamycin

Determination of the co-production of the globomycin, arylomycin and siamycin antibiotics in R5 crude extracts was performed with a Thermo Scientific Dionex UltiMate 3,000 UHPLC system (Thermo Scientific, United States) equipped with a DAD detector and TSQ quantis triple quadrupole MS detector coupled with an electrospray ionization source (ESI) using a Thermo Hypersil GOLD C18 column (100 × 2.1 mm, 3 μm) with a flow rate of 0.3 mL min-1. Mobile phase and elution conditions are the same as described below, with a column temperature of 35°C. Detection wavelength was from 210 to 600 nm and mass scanning range was from 200 to 2,500 *m/z*. Positive ionization spray voltage was set to 3,500 V, ion transfer tube temperature was 350°C with a vaporizer temperature of 400°C.

## Data Availability

The complete genome sequence of Streptomyces sp. VB1 was deposited in GenBank under the accession numbers CP110539 and CP110540, for chromosome and plasmid, respectively. Metabolomics data analysis can be found in https://gnps.ucsd.edu/ProteoSAFe/status.jsp?task=804381f36e904c53aa4eaf3412eb670e.

## Footnotes

^1^ https://ccms-ucsd.github.io/GNPSDocumentation/

^2^ https://ccmsucsd.github.io/NPSDocumentaton/molnetenhacer/

^3^Deposited as a Jupyter Notebook at https://github.com/LeonardoZamoraLeiva/gfamheatmap
