# Supplementary material for: Unveiling metabolo-genomic insights of potent antitumoral and antibiotic activity in Streptomyces sp. VB1 from Valparaíso Bay
Source: Front Microbiol. 2024 Oct 2;15:1463911. doi: 10.3389/fmicb.2024.1463911 (PMC11479970; doi:10.3389/fmicb.2024.1463911)
Supplement: Supplementary file 1 [file Table_1.DOCX]

Supplementary Material


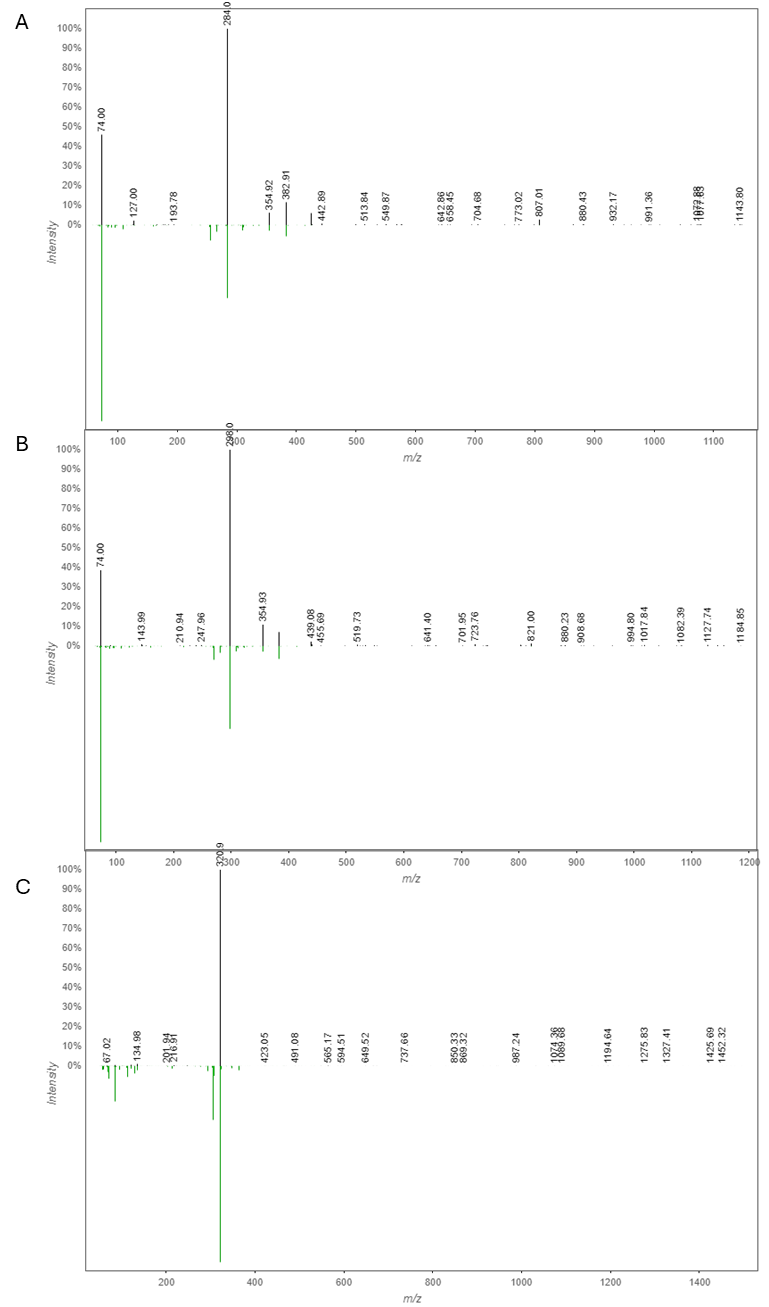


**Supplementary Figure S1.** Mirror plot comparison of A) Arylomycin A2 B) Arylomycin A4 C) Daunorubicin. The upper spectrum (black) represents the reference spectrum from the GNPS library, while the lower spectrum (green) corresponds to spectrum obtained from the crude extract. The comparison illustrates the similarity between the two spectra, confirming the presence of these bioactive compounds in the crude extract.

.
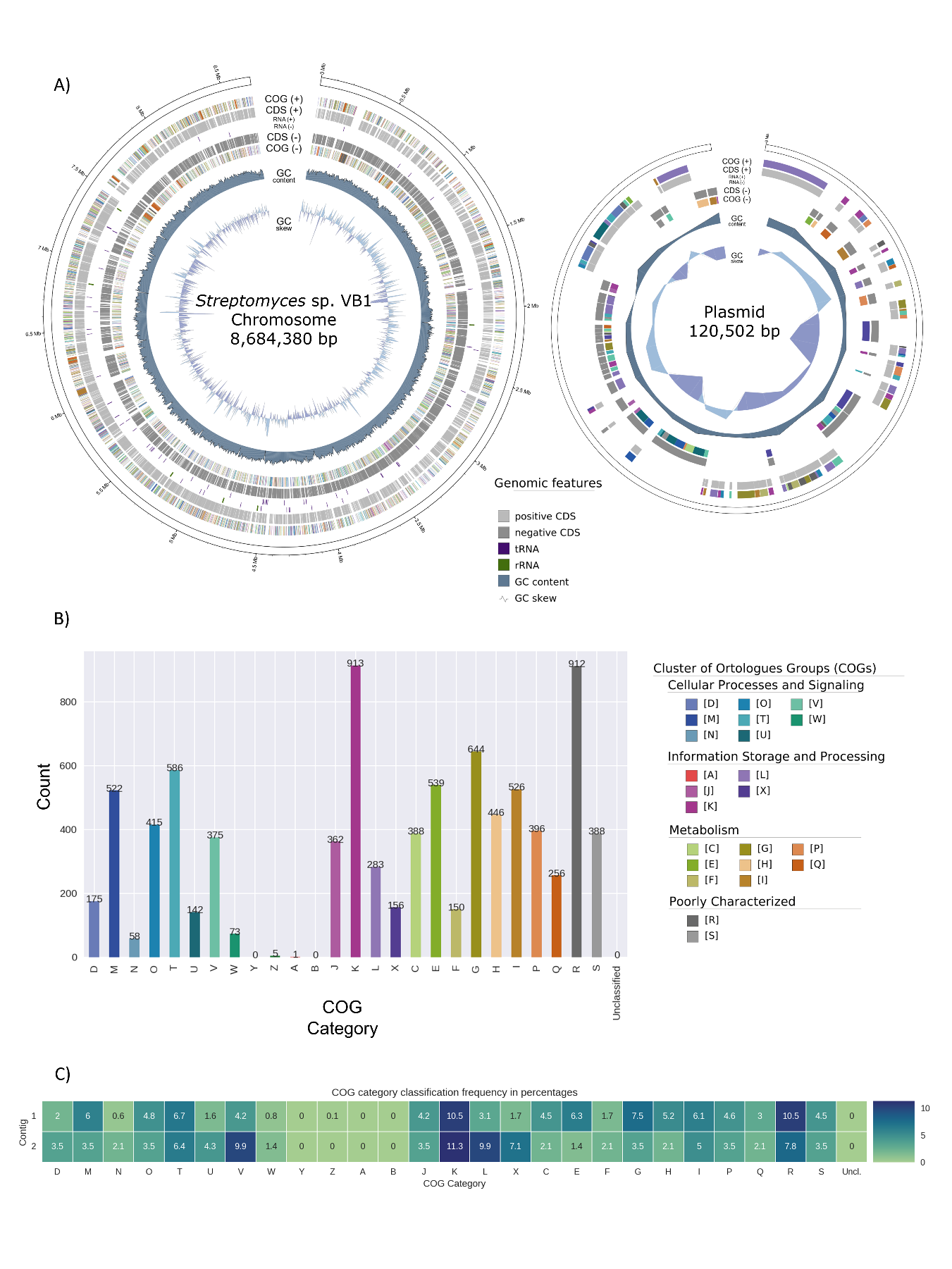


**Supplementary Figure 2.** Circular map and COG genes classification of *Streptomyces* sp. VB1 genome**.** A) Genome map of the chromosome and plasmid obtained for the *Streptomyces* sp. VB1 genome. The outer scale is numbered clockwise in megabases. The corresponding description for each circle track within the ideogram is shown at the intersection of both ends. Note that circular representation does not necessarily reflect the nature of the replicon itself. B) Classification of genes into COG categories, showing the count of genes for each. C) COG classification frequencies by percentage: Contig 1 = Chromosome, Contig 2 = Plasmid.


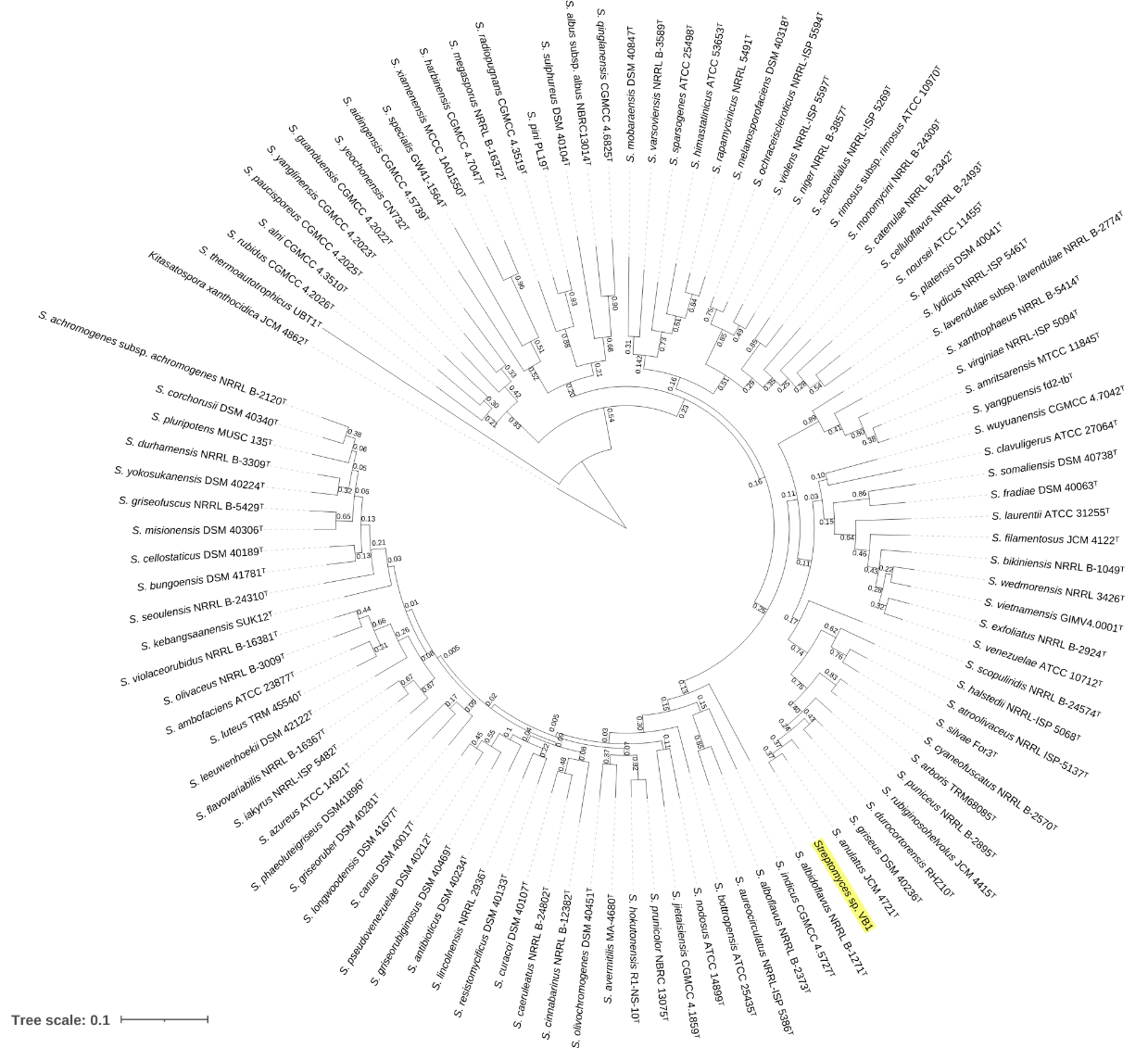


**Supplementary Figure 3.** Phylogenomic inference of *Streptomyces* evolutionary relationships. Phylogeny of *Streptomyces* type strains (dataset of n=105 including *Streptomyces* sp. VB1 and *Kitasatospora xanthocidica* JCM 4862 as outgroup, Table S5) inferred from 763,085 genes assigned to orthogroups (orthologous genes translated to protein sequences). FastTree was used for approximate maximum-likelihood tree inference. Bar, evolutionary distance, considering 0.1 substitutions per amino acid position.


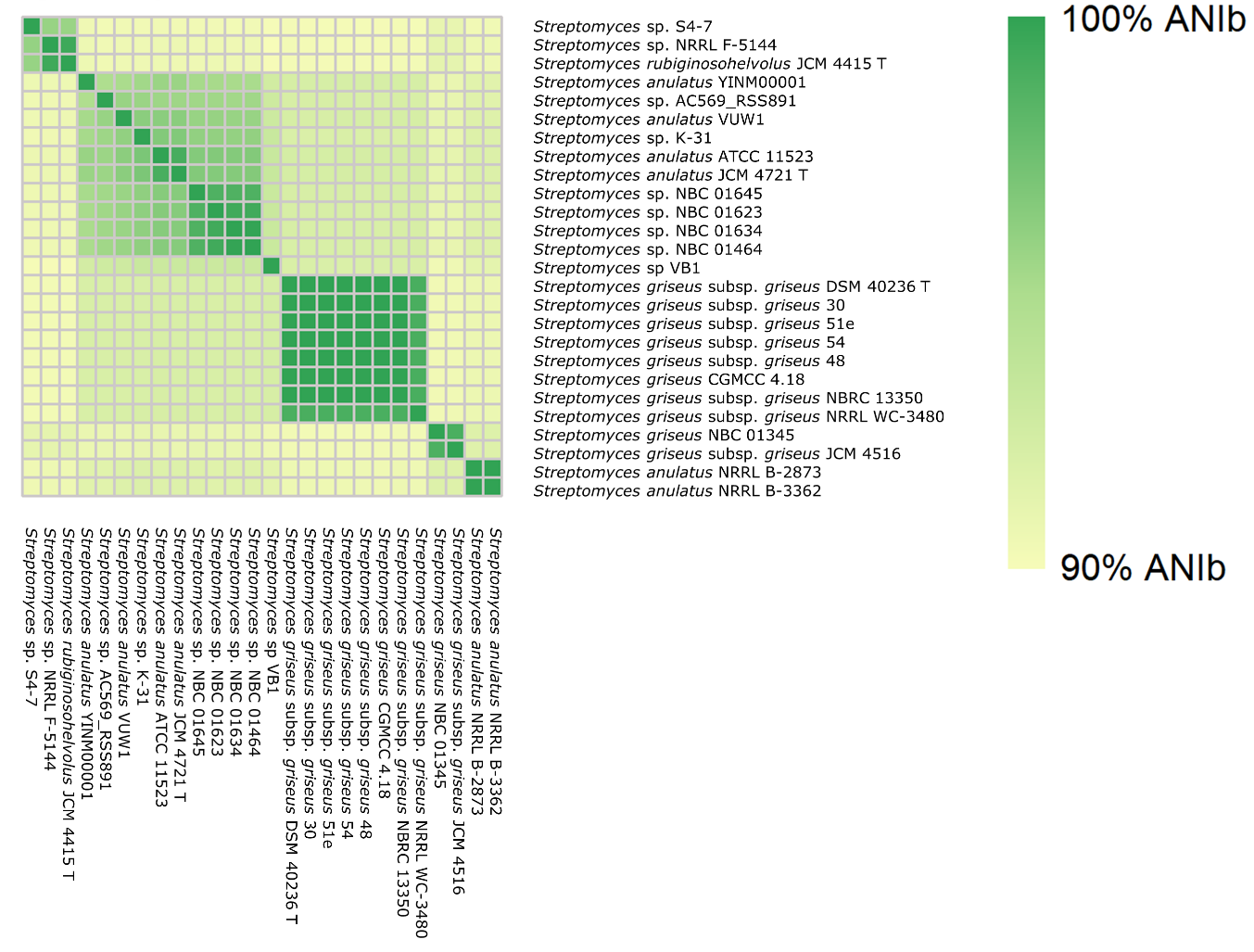


**Supplementary Figure 4.** ANIb of closest neighbours of *Streptomyces* sp. VB1. Percentage values are represented in a yellow to green colour scale.


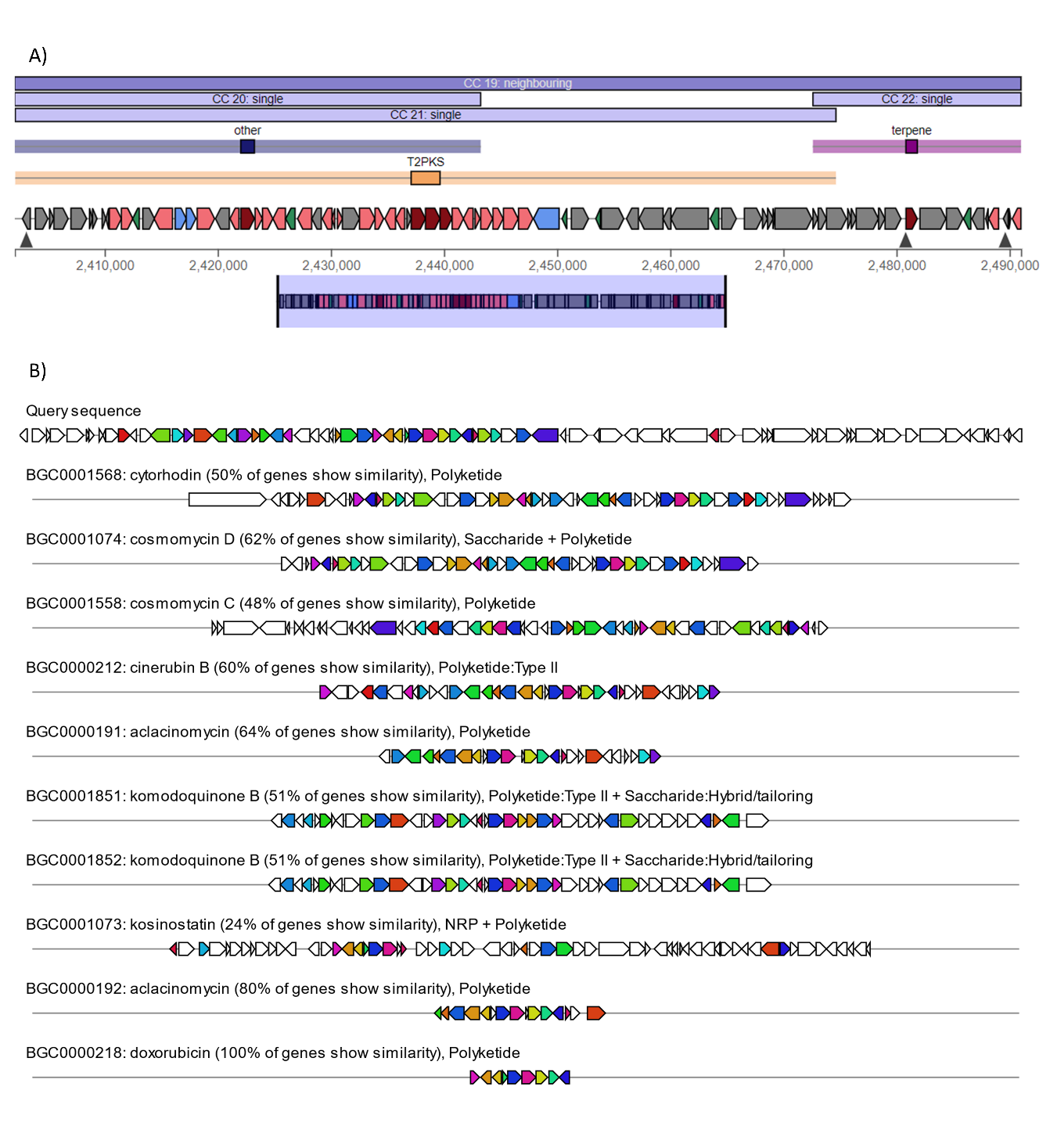


**Supplementary Figure 5**. BGC #15. A) Overview of BGC #15 of *Streptomyces* sp. VB1. B) Known Cluster Blast results for BGC #15 (query BGC), coding for the production of doxorubicin and depicting its similarity with genes from other BGCs.


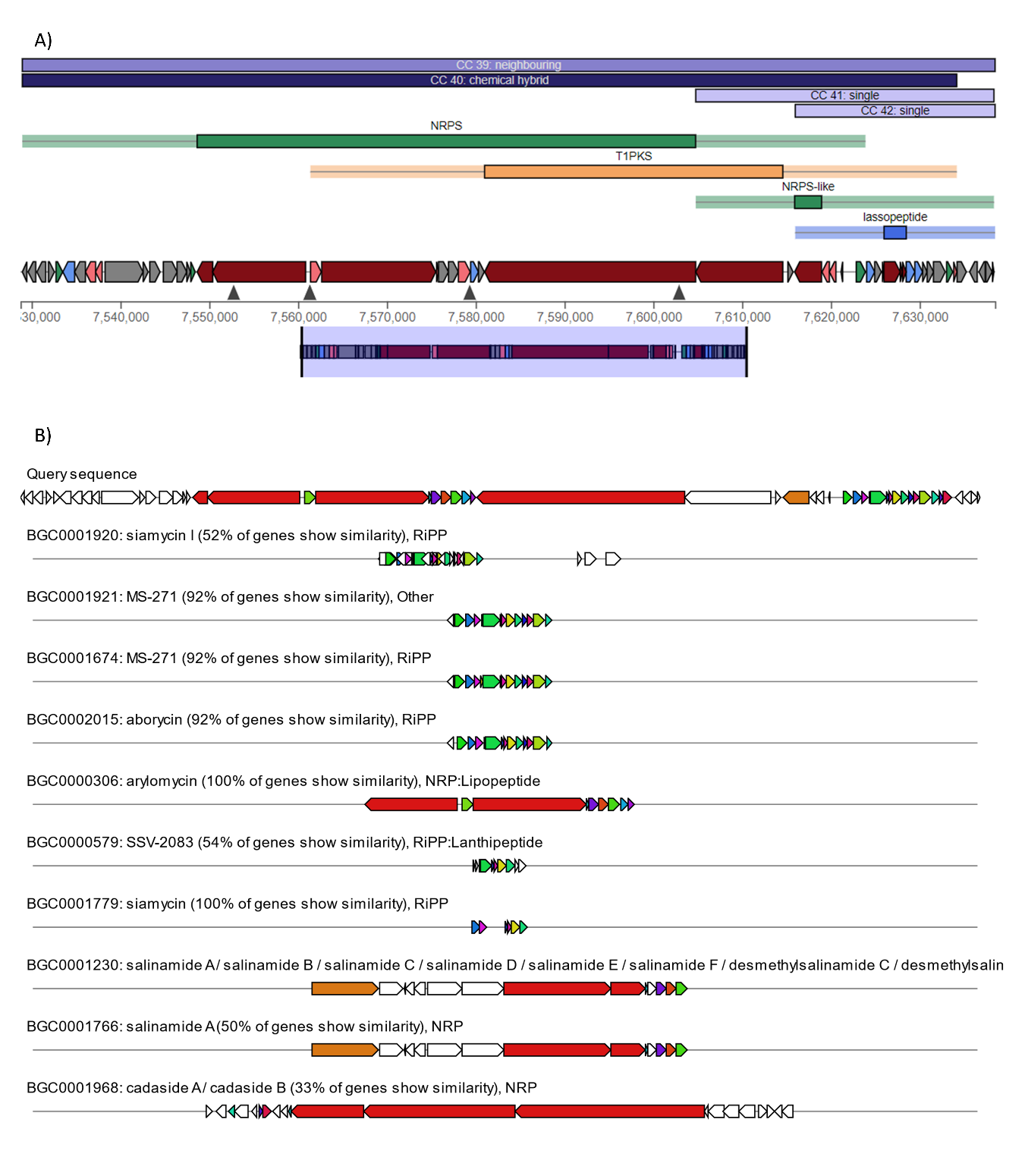


**Supplementary Figure 6.** BGC #28 A) Overview of BGC #28 of *Streptomyces* sp. VB1. B) Known Cluster Blast results for BGC #28, coding for the production of arylomycin and siamycin and depicting its similarity with other BGCs.


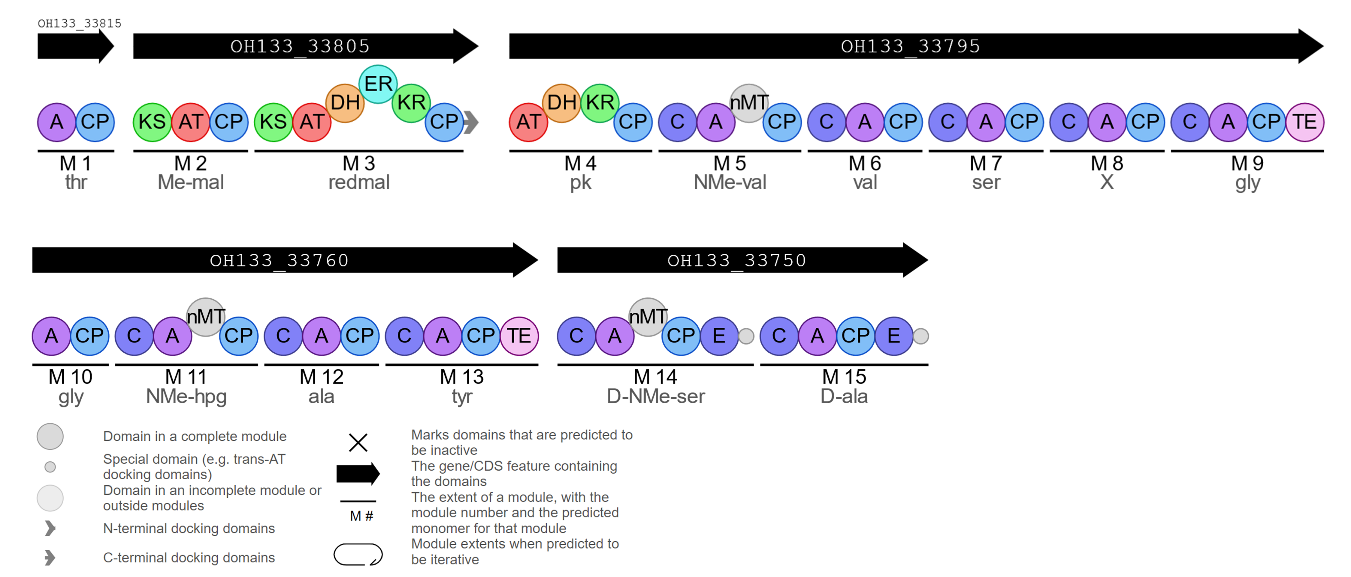


**Supplementary Figure 7.** Module view of BGC #28 obtained from AntiSMASH.


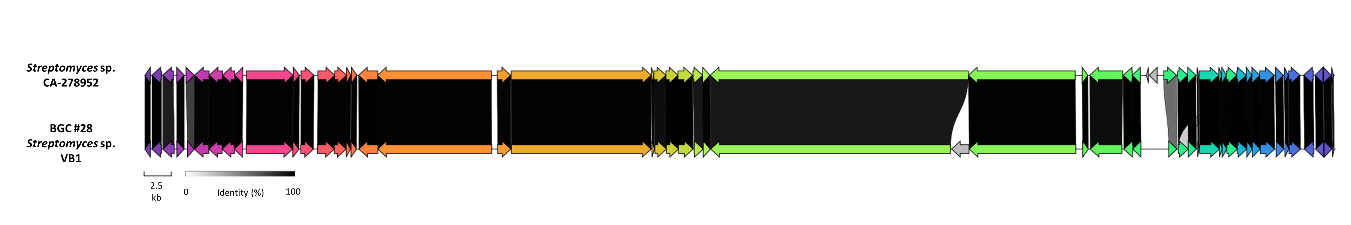


**Supplementary Figure 8.** Comparison of the superclusters from strains VB1 and CA-278952. Genes are coloured according to the presence of potential homologues in the other cluster, while for those in grey, no similar genes were found. Identity percentage between matching genes is shown in greyscale-coloured links.


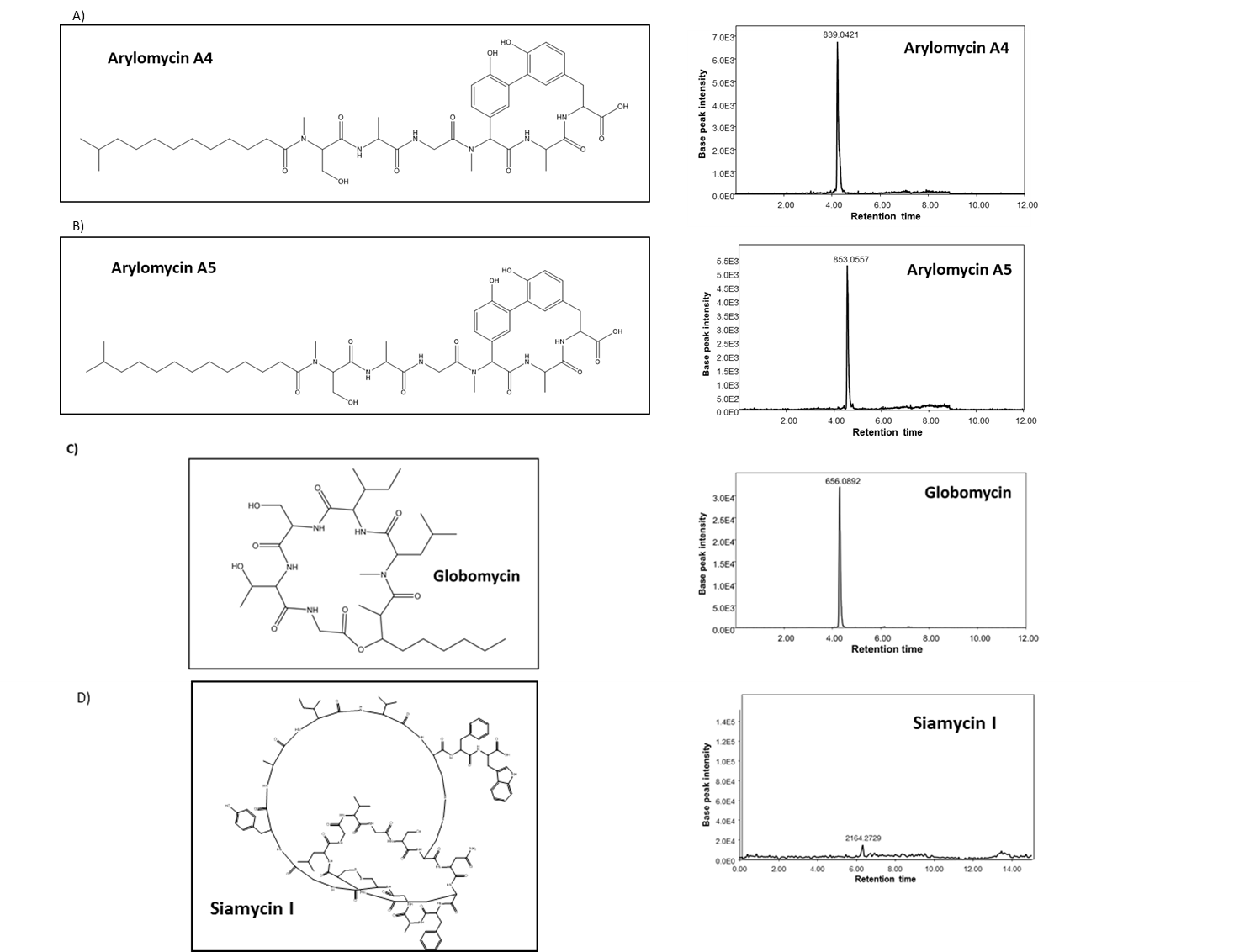


**Supplementary Figure 9.** Metabolic profiles of *Streptomyces* VB1. Positive EICs and molecular structure of A) Arylomycin A4; B) Arylomycin A5; and C) Globomycin, D) Siamycin I
